# Apigenin mitigates oxidative stress, neuroinflammation, and cognitive impairment but enhances learning and memory in aluminum chloride‐induced neurotoxicity in rats

**DOI:** 10.1002/alz.70223

**Published:** 2025-05-02

**Authors:** Ademola Adetokunbo Oyagbemi, Omowumi Moromoke Femi‐Akinlosotu, Adedunsola Adewunmi Obasa, Moses Semilore Ojo, Adeola Temitope Salami, Temitayo Olabisi Ajibade, Charles Etang Onukak, Olumayowa Olawumi Igado, Oluwaseun Olarenwaju Esan, Taiwo Olaide Oyagbemi, Adewumi Victoria Adeogun, Omolola Victoria Awoyomi, Joseph E. Ikokide, Ishmael Festus Jaja, Olufunke Eunice Ola‐Davies, Temidayo Olutayo Omobowale, Adebowale Bernard Saba, Oluwafemi Omoniyi Oguntibeju, Evaristus Nwulia, Momoh Audu Yakubu

**Affiliations:** ^1^ Department of Veterinary Physiology and Biochemistry Faculty of Veterinary Medicine University of Ibadan Ibadan Nigeria; ^2^ Developmental Neurobiology and Forensic Anatomy Unit Department of Anatomy College of Medicine University of Ibadan Ibadan Nigeria; ^3^ Neuroscience Unit Department of Veterinary Anatomy Faculty of Veterinary Medicine University of Ibadan Ibadan Nigeria; ^4^ Development of Physiology Faculty of Basic Medical Sciences College of Medicine University of Ibadan Ibadan Nigeria; ^5^ Department of Veterinary Medicine Faculty of Veterinary Medicine University of Ibadan Ibadan Nigeria; ^6^ Federal College of Animal Health and Production Technology Ibadan Nigeria; ^7^ Department of Theriogenology Faculty of Veterinary Medicine University of Ibadan Ibadan Nigeria; ^8^ Department of Livestock and Pasture Science Faculty of Science and Agriculture University of Fort Hare Alice South Africa; ^9^ Department of Agriculture and Animal Health University of South Africa, Roodepoort Johannesburg South Africa; ^10^ Department of Veterinary Pharmacology and Toxicology Faculty of Veterinary Medicine University of Ibadan Ibadan Nigeria; ^11^ Phytomedicine and Phytochemistry Group Department of Biomedical Sciences Faculty of Health and Wellness Sciences Cape Peninsula University of Technology Bellville South Africa; ^12^ College of Medicine Department of Psychiatry and Behavioral Sciences Howard University Hospital, Howard University Washington District of Columbia USA; ^13^ Department of Environmental & Interdisciplinary Sciences College of Science Engineering & Technology Vascular Biology Unit Center for Cardiovascular Diseases COPHS, Texas Southern University Houston Texas USA

**Keywords:** Apigenin, cognitive impairment, neurotoxicity, oxidative stress

## Abstract

**INTRODUCTION:**

Aluminum chloride (AlCl_3_) exposure has been linked to neurotoxicity in various animal models, presenting significant concern to human health due to its potential implications in neurodegenerative diseases. Aluminum chloride is a widely recognized neurotoxin and has been used as an animal model of Alzheimer's disease via mechanisms linked with oxidative stress and inflammation. The study investigated the potential ameliorative effect of apigenin on AlCl_3_‐induced neurotoxicity in rats.

**METHODS:**

Forty adult male Wistar rats were randomly divided into four different groups – control, AlCl_3_ (100 mg/kg), apigenin (50 mg/kg) plus AlCl_3_, and apigenin (50 mg/kg) alone administered orally for 14 days.

**RESULTS:**

Our findings revealed AlCl_3_ exposure induced significant neurobehavioral deficits, oxidative stress, neuroinflammation, and loss of the Purkinje cell layer of the cerebellum. Treatment with apigenin attenuated neuroinflammation and enhanced learning and memory with significant improvement in recognition index.

**DISCUSSION:**

Apigenin demonstrates promising ameliorative effects against AlCl_3_‐induced neurotoxicity in rats.

**Highlights:**

- Aluminum chloride toxicity caused significant reduction in learning, exploration, and memory.
- Aluminum chloride toxicity induced neurotoxicity, increased biomarkers of oxidative stress, neuroinflammation, and precipitated cognitive impairment.
- Apigenin improved brain antioxidant, enhanced learning, exploration, and memory.

## Graphical abstract

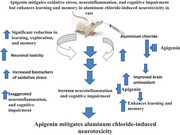

## 1 BACKGROUND

Neurotoxicity is considered to have an adverse impact on the chemistry, structure, or function of the central or peripheral nervous system caused by physical or chemical substances.^1^, ^2^ The toxic actions of chemical substances ultimately impact negatively on these nervous systems, which might affect locomotor function, speech, exploration, learning, and memory.

**FIGURE 1:**
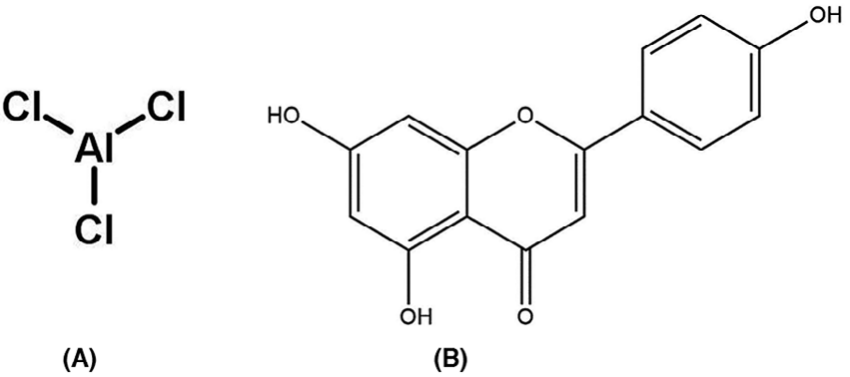
(A) Structure of apigenin (4′,5,7,‐trihydroxyflavone). (B) Structure of aluminum chloride (AlCl_3_).

Potentially harmful to the nervous system, aluminum chloride (AlCl_3_), as indicated in Figure 1A, can oxidatively damage a variety of cellular indicators following long‐term exposure.^3^ Aluminum chloride has the potential to worsen oxidative brain damage, increase amyloid beta (Aβ) deposition, and cause inflammation.^4^, ^5^ Recently, reports from various laboratories have documented the use of AlCl_3_ toxicity as an experimental model of Alzheimer's disease (AD).^6^, ^7^ Numerous investigations have documented the neuroprotective properties of several agents proven to mitigate neurotoxicity induced by AlCl_3_ toxicity.^8^, ^9^ For instance, it has been reported that ononin and baicalein have neuroprotective properties against AD precipitated by AlCl_3_ toxicity.^10^, ^11^ These compounds are natural isoflavone glycoside and flavonoids derived from the roots, stems, rhizome, *Oroxylum indicum* (L.) Kurz, and *Scutellaria baicalensis* Georgi.^12^, ^13^

Also, experimental animals intoxicated with AlCl_3_ have been shown to benefit from the neuroprotective action of *Xylopia parviflora* and syringic acid.^14^, ^15^ Due to its unwavering high affinity for transferrin receptors, aluminum can readily cross the blood‐brain barrier (BBB) and accumulate in the brain.^16^, ^17^ Aluminum primarily accumulates in the frontal cortex and hippocampal regions of the brain with elevated levels of inflammatory cytokines and oxidative stress indicators.^18^, ^19^, ^20^ Neurotransmission systems of the brain are readily disturbed by the combination of AlCl_3_ and sodium fluoride toxicity.^21^

AD is caused by a combination of environmental and hereditary factors. However, families with AD are thought to benefit from genetic mutations linked to both the metabolism and expression of amyloid precursor protein (APP), as no evidence has been found that APP mutations impair the physiological function of APP.^22^, ^23^ Furthermore, prolonged exposure to environmental metals can cause neurological diseases like AD.^3^, ^24^, ^25^ Aluminum can specifically accelerate the rate of diffusion across the BBB.^18^ Consequently, there is evidence of both molecular and systemic consequences from exposure to aluminum toxicity. Chronic aluminum poisoning has been documented for its neurotoxic effects.^26^, ^27^

Apigenin (4′,5,7‐trihydroxyflavone) is a dietary flavonoid (Figure 1B) present in a broad range of fruits, vegetables, and medicinal herbs.^28^ Its pharmacological action on neurological illnesses is aided by its capacity to cross the BBB.^29^ Apigenin has been found to regulate cyclic adenosine monophosphate (cAMP) response element‐binding protein (CREB) brain‐derived neurotrophic factor (BDNF) cAMP‐CREB‐BDNF and N‐methyl‐D‐aspartate (NMDA) receptors signaling cascade that are crucial for neuronal survival, synaptic plasticity, cognitive function, and mood behavior.^30^, ^31^

Apigenin as a flavonoid has been utilized to mitigate AD via reduction in neuroinflammation, protecting neurons from oxidative stress and enhancing neuronal survival and synaptic plasticitity.^32^ Dietary supplementation of apigenin has been formulated into tablets, capsules, solid dispersions, co‐crystals, inclusion complexes, and nano formulations with significant health benefits.^28^ Other research findings have reported the mitigating action of apigenin by lowering the build‐up of Aβ plaque in AD progression.^33^ Recently, apigenin administration was found to offer neuroprotection by upregulating BDNF, phosphorylated extracellular signal‐regulated kinase, and CREB.^34^ *In vitro, in vivo*, and in silico analyses revealed that flavonoids such as apigenin could effectively inhibit acetylcholinesterase activity and, therefore, could be effectively utilized as a potential therapeutic agent for cognitive dysfunction.^35^ Farbin and his colleagues reported the application of apigenin in autism spectrum disorder (ASD)‐like phenotype through reduction in oxidative stress, inflammation, and enhancement of cognitive function in the prefrontal cortex.^36^

Based on the available information on the neuroprotective actions of apigenin and its ability to readily cross the BBB, we hypothesized that apigenin would improve cognitive decline and enhance learning and memory in AlCl_3_‐induced neurotoxicity. Additionally, we explored the effects of AlCl_3_ toxicity on cerebellar Purkinje cell layer loss and cognitive impairment by employing a novel object recognition test as one of the gold standards for cognitive impairment.

## 2 METHODS

### 2.1 Experimental animals

Twenty‐four male Wistar strain rats were used in this study. They were purchased from the Animal House of the Faculty of Veterinary Medicine, University of Ibadan, Ibadan, Nigeria. In addition, they were randomly selected, acclimated to laboratory conditions for 7 days, and fed standard feed and water *ad* *libitum*. The weight range of the rats was 100 to 120 g at the commencement of experiments. All the experimental animals received humane care according to the criteria outlined in the Guide for the Care and the Use of Laboratory Animals, and ethics regulations were followed in accordance with the 1996 National Institutes of Health national and institutional guidelines for the protection of animal welfare during experiments.

**RESEARCH‐IN‐CONTEXT**

1. **Systematic review**: The authors sought to explore the impact  of aluminum chloride toxicity on the induction of oxidative stress, learning, exploration, and memory performance evaluation in AD animal models using aluminum chloride‐induced neurotoxicity. There have been several publications featuring (AD) animal models using aluminum chloride neurotoxicity, but there is a paucity of information on the supplementation of apigenin against aluminum chloride‐induced neurotoxicity.
2. **Interpretation**: Our findings led to our research hypothesis regarding whether apigenin supplementation improved cognitive impairment.
3. **Future directions**: (1) We aim to continue to evaluate the novel mechanism of action of apigenin on cognitive impairment in animal models of AD. (2) Bench‐to‐bedside application of apigenin in enhancing learning and memory should be explored.

### 2.2 Study design

The study was divided into four groups comprising six animals each. The rats in the first group (Group 1) were administered normal saline and served as the control. The rats in Group 2 were administered aluminum chloride at 100 mg/kg.^31^ The rats in Group 3 were administered aluminum chloride at 100 mg/kg and apigenin at 50 mg/kg.^37^ The rats in Group 4 were administered apigenin 50 mg/kg. All administrations were done by oral gavage using bulb steel needles. The administration period lasted for 14 consecutive days.

### 2.3 Animal care

The experimental protocol was implemented entirely in conformity with the guidelines of the Animal Research Review Panel for the care and use of laboratory animals. Ethical approval for the study and consent for the use of animals was obtained from University of Ibadan, Animal Care and Use Research Committee (UI‐ACUREC) with approval number UI‐ACUREC/059‐0324/28.

### 2.4 Novel object recognition test

The novel object recognition test (NORT) is used to evaluate cognition in rats as it relates to different aspects of learning and memory.^38^ A general description for the test is as follows. In an open field box, the rats are first familiarized with two identical objects (habituation phase). Twenty‐four hours later (test phase), one of the objects was replaced with a new object and the rats were allowed to explore both objects for 5 min. The time spent exploring each object was recorded.

### 2.5 Brain sample collection and preparation

At termination of the study, rats were humanely euthanized, and thereafter, brain tissues were harvested for biochemical assay. The rats were anesthetized with 0.1 mL of xylazine/ketamine (v/v), while overdose of xylazine/ketamine was used for euthanasia.

### 2.6 Brain post‐mitochondrial fraction preparation

Brain samples were quickly excised, rinsed, blotted with filter paper, weighed, chopped into bits, and homogenized with homogenizing buffer (0.1 M phosphate buffer, pH 7.4) using a Teflon homogenizer. The homogenate obtained was centrifuged at 10,000 rpm for 10 mins with a cold centrifuge at −4°C to obtain post‐mitochondrial fractions (PMFs). The PMFs obtained were used for the biochemical assays.

### 2.7 Determination of brain antioxidant defense status

Superoxide dismutase (SOD) assay was carried out by the method of Misra and Fridovich^39^ with slight modification as described earlier.^40^, ^41^ Glutathione peroxidase (GPx) activity was also measured according to Beutler et al.^42^ Glutathione S‐transferase (GST) was estimated by the method of Habig et al.^43^ using 1‐chloro‐2,4‐dinitrobenzene (CDNB) as substrate, and the reduced glutathione (GSH) content was estimated by the method of Ellman.^44^

### 2.8 Determination of brain markers of oxidative stress

Hydrogen peroxide (H_2_O_2_) content was determined using the method described by Wolf.^45^ Malondialdehyde (MDA) content as a product of lipid peroxidation was determined using the method described by Varshney and Kale.^46^ The acetylcholinesterase (AChE) activity was evaluated with the method of Dingova et al.^47^ Nitric oxide level was determined using the method described by Olaleye et al.,^48^ while serum total protein was determined by Biuret's method, as described by Gornal et al.^49^

### 2.9 Histological evaluation

Mid‐sagittal cut of the whole brain was harvested, while the left hemisphere was sectioned for histological evaluation and fixed in 10% formalin. The fixed tissues were immersed in paraffin wax, sectioned, and mounted on slides for histopathological evaluation after staining with Nissl, as previously described by Drury and Wallington.^50^

### 2.10 Statistical analysis

All values were expressed as mean ± standard deviation. One‐way ANOVA with Turkey's post hoc test was carried out, with *p* values < .05 considered statistically significant.^51^

## 3 RESULTS

**FIGURE 2:**
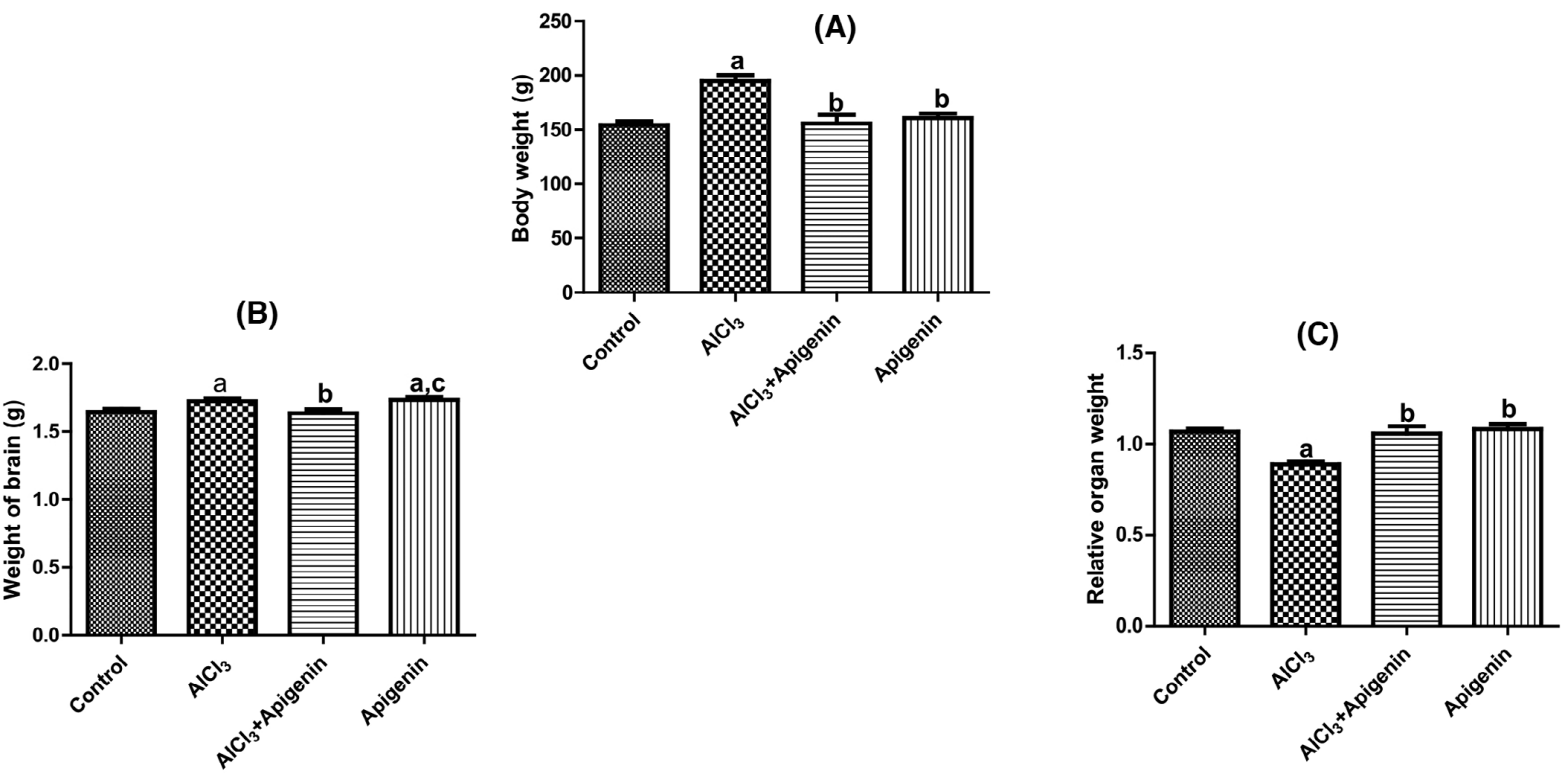
Effects of aluminum chloride‐induced neurotoxicity on body weight, brain weight, and relative brain weight. Letter a indicates significant difference compared with control, while letter b indicates significant difference compared with AlCl_3_, and letter c indicates significant difference compared with AlCl_3_ + apigenin. Mean ± SD (*n* = 6).

**FIGURE 3:**
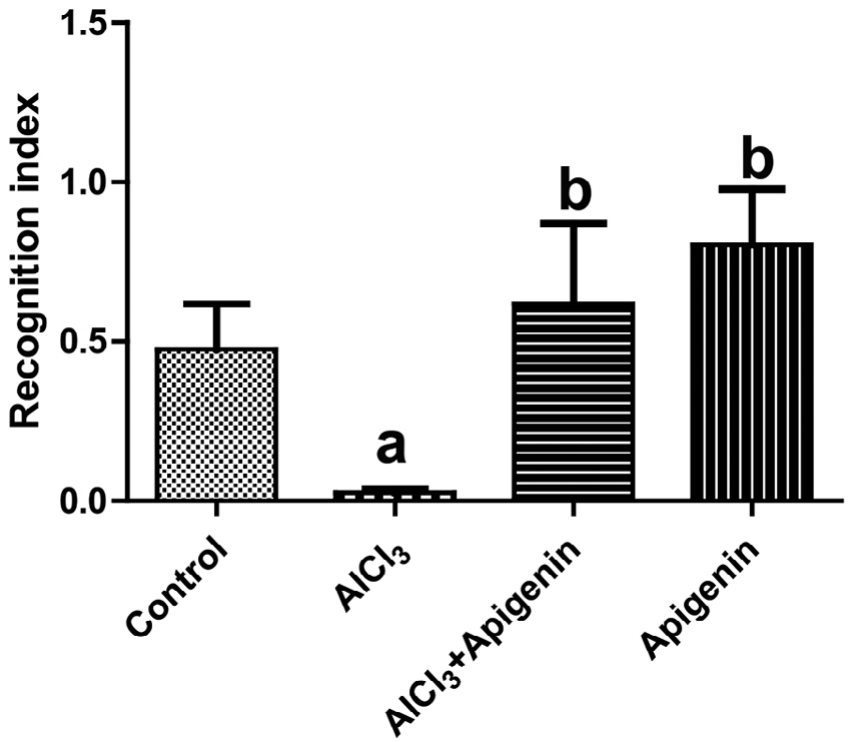
Effects of aluminum chloride‐induced neurotoxicity on recognition index. Letter a indicates significant difference compared with control, while letter b indicates significant difference compared with AlCl_3_. Mean ± SD (*n* = 6).

The results of this study showed that AlCl_3_‐induced neurotoxicity caused a significant (*p *< 0.05) increase in the body weight of the group administered AlCl_3_ alone, while there was a significant (*p *< 0.05) decrease in the body weights of the group co‐administered AlCl_3_ and apigenin, and a decrease in the body weight of the group administered apigenin alone (Figure 2A). The study's results also showed that AlCl_3_‐induced neurotoxicity caused a significant (*p *< 0.05) reduction in the brain weight of the group administered only AlCl_3_ in comparison to the control group (Figure 2B). The brain weight of the group co‐administered AlCl_3_ and apigenin was significantly (*p *< 0.05) lower in comparison to the group administered AlCl_3_. The brain weight of the group administered apigenin only was significantly (*p *< 0.05) higher compared to the group administered AlCl_3_ only and the group co‐administered AlCl_3_ and apigenin (Figure 2B). The study's results further showed that AlCl_3_‐induced toxicity caused a significant (*p *< 0.05) reduction in the relative brain weight of the group administered AlCl_3_ only compared to the control group (Figure 2C). However, there was a significant (*p *< 0.05) increase in the relative organ weight of the groups administered apigenin compared to the group administered AlCl_3_ (Figure 2C). In the neurobehavioral assessment, AlCl_3_ significantly (*p *< 0.05) reduced the recognition index, while rats co‐administered apigenin had a significantly improved recognition index (Figure 3).

**FIGURE 4:**
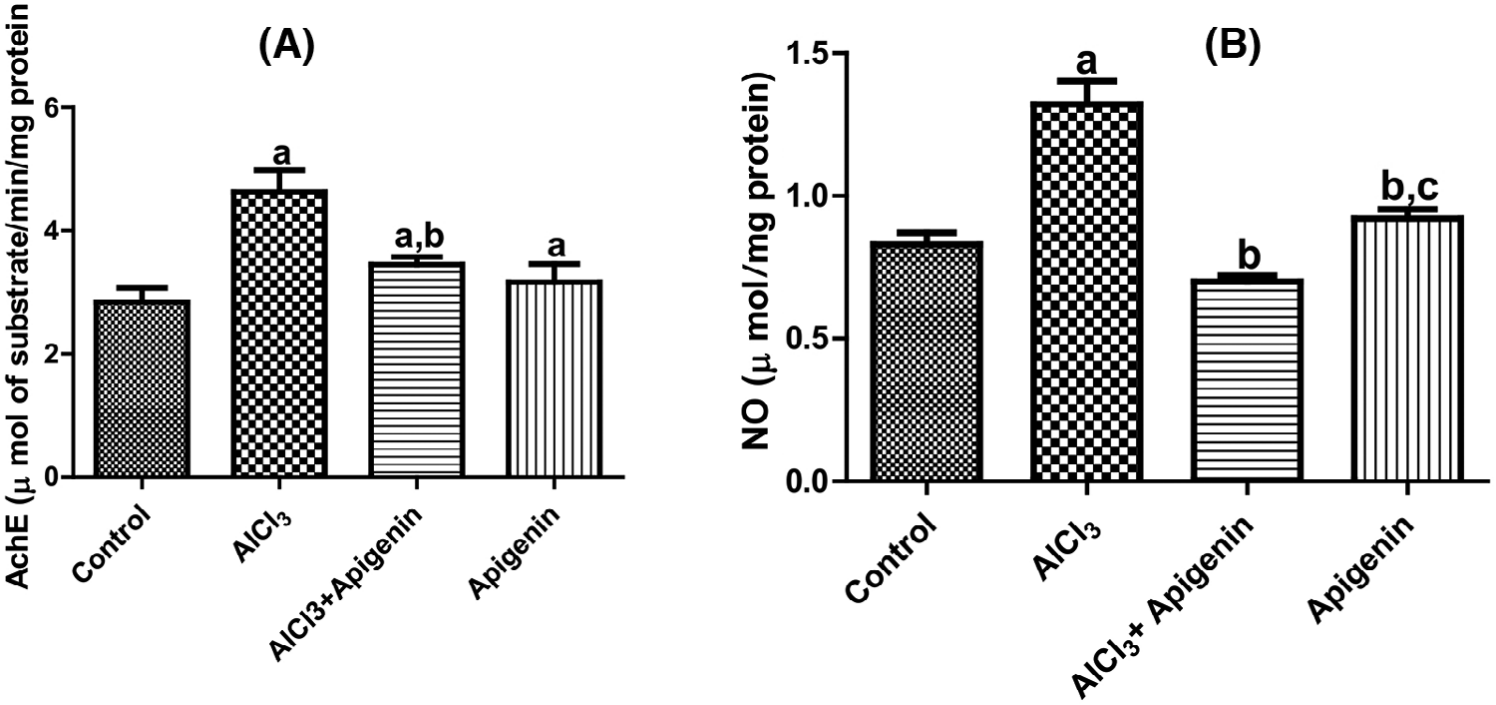
Effects of aluminum chloride‐induced neurotoxicity on brain nitric oxide (NO) level and acetylcholinesterase (AChE) activity. Letter a indicates significant difference compared with control. letter b indicates significant difference compared with AlCl_3_, while letter c indicates significant difference compared with AlCl_3_ + apigenin. Mean ± SD (*n* = 6).

In this study, the toxic effects of AlCl_3_ on brain acetylcholinesterase activity were assessed. It was shown that AlCl_3_ toxicity caused a significant (*p *< 0.05) increase in AChE activity in all treated groups compared to control (Figure 4A). We also observed a significant (*p *< 0.05) reduction in AChE activity in rats treated with apigenin and those that received only apigenin relative to untreated rats (Figure 4A). Nitric oxide is another biomarker of neuroinflammation and nitrosative stress. In Figure 4B, we demonstrate that AlCl_3_ toxicity caused a significant (*p *< 0.05) increase in NO contents compared with the control. There was also a significant (*p *< 0.05) reduction in the content of NO in animals that received both AlCl_3_ and apigenin as well as those that received only apigenin compared with rats administered AlCl_3_ alone (Figure 4B). There was also a significant (*p *< 0.05) increase in the NO of rats only on apigenin compared with rats that received both AlCl_3_ and apigenin (Figure 4B).

**FIGURE 5:**
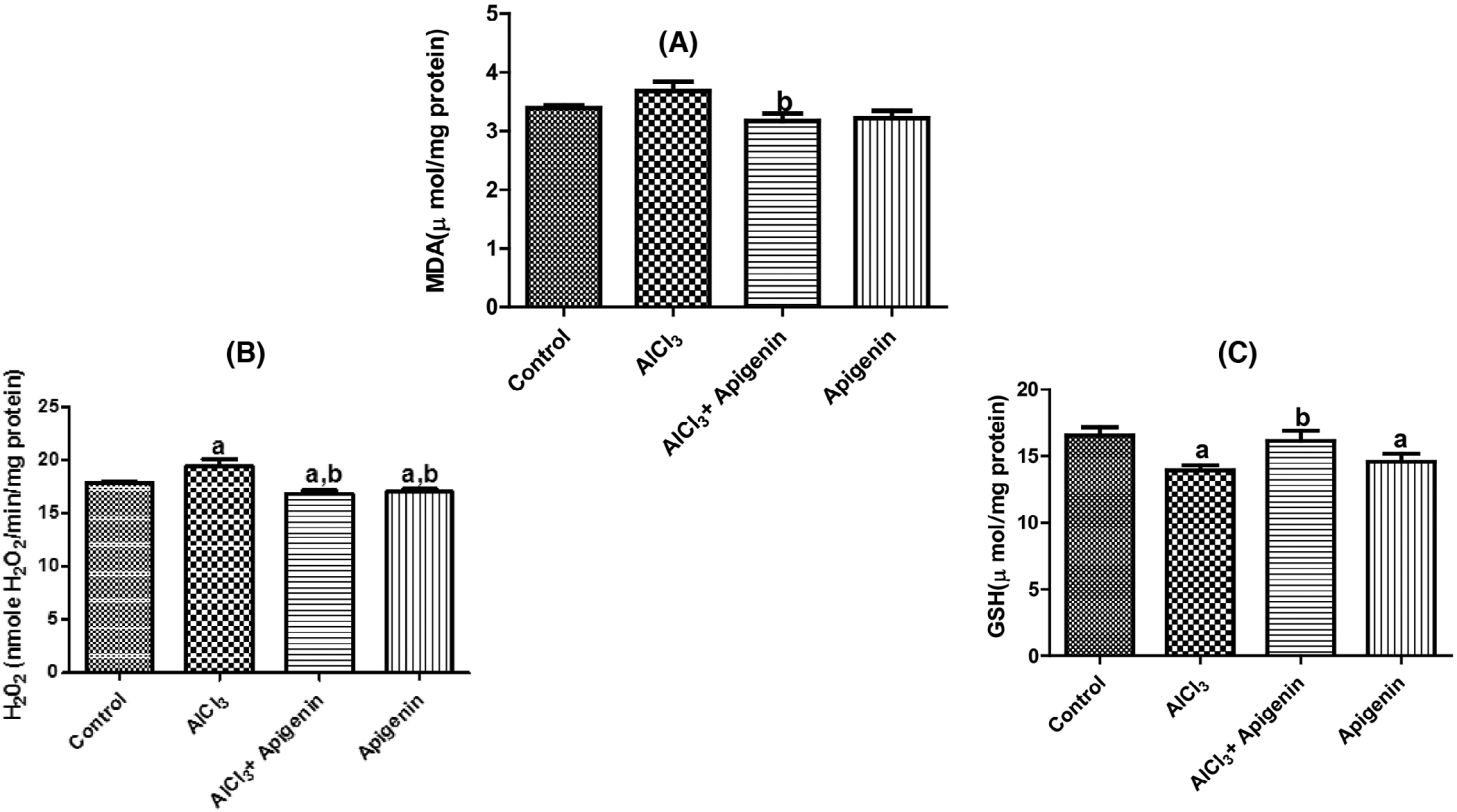
Effects of aluminum chloride‐induced neurotoxicity on brain malondialdehyde (MDA) levels and content of hydrogen peroxide generation. Letter a indicates significant difference compared with control, while letter b indicates significant difference compared with AlCl_3_. Mean ± SD (*n* = 6).

Oxidative stress biomarkers are essential components of toxicity assay associated with AlCl_3_‐induced neurotoxicity. Brain MDA content, hydrogen peroxide (H_2_O_2_) generation, and reduced glutathione (GSH) were determined. The study revealed a significant (*p *< 0.05) increase in MDA content in rats intoxicated with AlCl_3_ in comparison to the control (Figure 5A). Interestingly, co‐administration of AlCl_3_ + apigenin caused a significant reduction in MDA content compared to untreated AlCl_3_ rats. Figure 5B demonstrates that AlCl_3_ toxicity caused a significant (*p *< 0.05) increase in H_2_O_2_ generation compared to the control group. Furthermore, a significant (*p *< 0.05) decrease in H_2_O_2_ generation was observed following the co‐administration of AlCl_3_ + apigenin and apigenin only compared with the control and AlCl_3_‐intoxicated rats (Figure 5B). In this study, AlCl_3_ neurotoxicity on brain reduced glutathione (GSH) was assessed (Figure 5C). The results showed that there was a significant (*p *< 0.05) decrease in GSH levels in rats not treated with AlCl_3_ in comparison to the control. However, a significant improvement in the GSH content was recorded in rats administered AlCl_3_ + apigenin relative to rats not treated with AlCl_3_ (Figure 5C).

**FIGURE 6:**
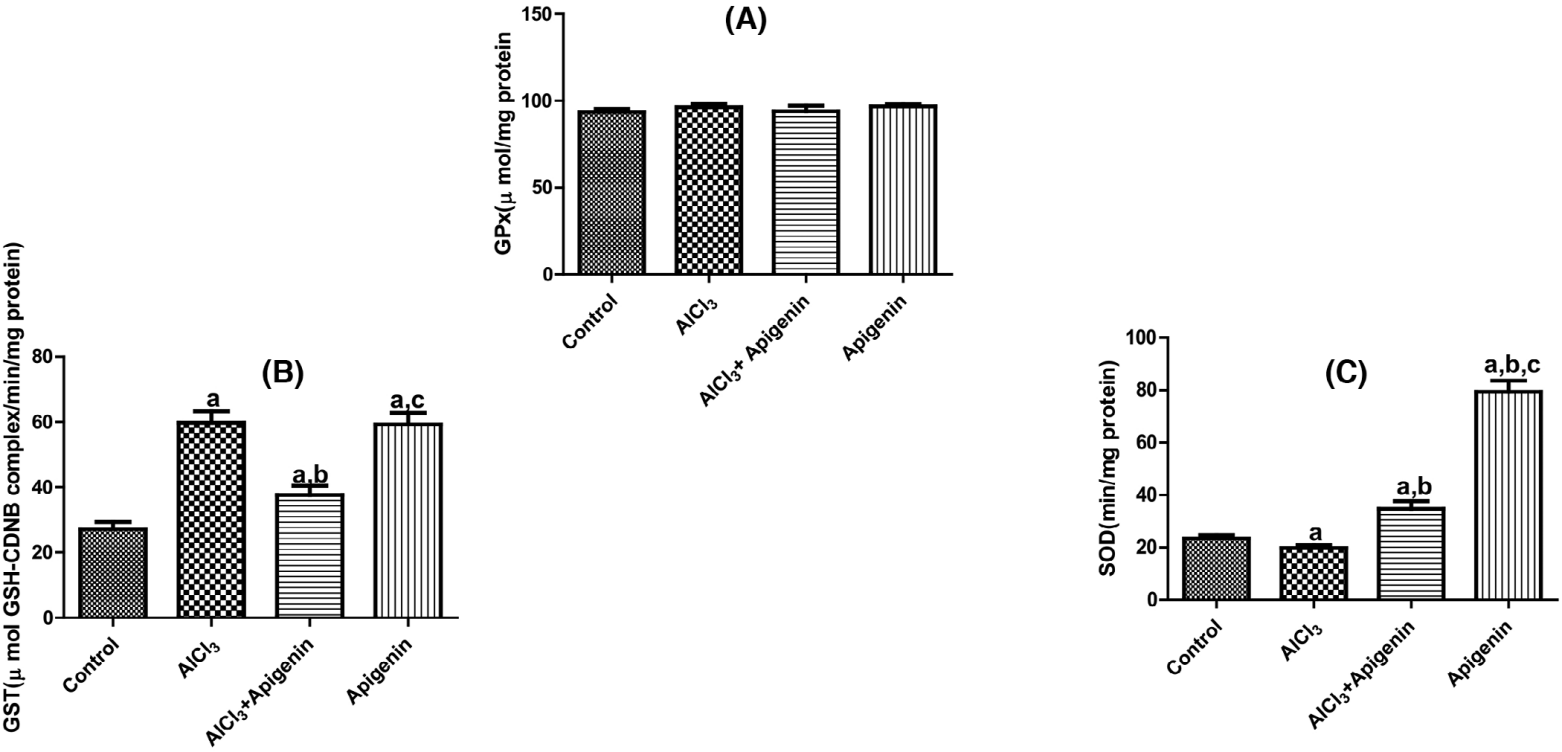
Effects of aluminum chloride‐induced neurotoxicity on brain glutathione S‐transferase and superoxide dismutase activity. Letter a indicates significant difference compared with control. Letter b indicates significant difference compared with AlCl_3_, while letter c indicates significant difference compared with AlCl_3_ + apigenin. Mean ± SD (*n* = 6).

The activity of GPx was assessed following AlCl_3_‐induced neurotoxicity (Figure 6A). From the result obtained, there was no statistically significant (*p* > 0.05) difference in GPx activity across all treatment groups compared to the control (Figure 6A). From this study, Figure 5B shows that there was a significant (*p *< 0.05) increase in the GST activity of rats administered AlCl_3_, AlCl_3_ + apigenin, and apigenin compared with the control group. Similarly, a significant (*p *< 0.05) increase in the GST activity of rats treated with AlCl_3_ + apigenin compared with those treated with AlCl_3_ was recorded (Figure 5B). Furthermore, a significant (*p *< 0.05) increase in the GST activity of rats administered apigenin was observed compared with the AlCl_3_ + apigenin group (Figure 6B). In another experiment, we recorded that AlCl_3_‐induced neurotoxicity significantly decreased (*p *< 0.05) SOD activity compared to the control and other treatment groups (Figure 6C). However, in rats receiving co‐administration of AlCl_3_ with apigenin and those on only apigenin had a significantly improved SOD activity compared with the control and AlCl_3_‐untreated groups, indicating the antioxidant capacity of apigenin (Figure 6C).

**FIGURE 7:**
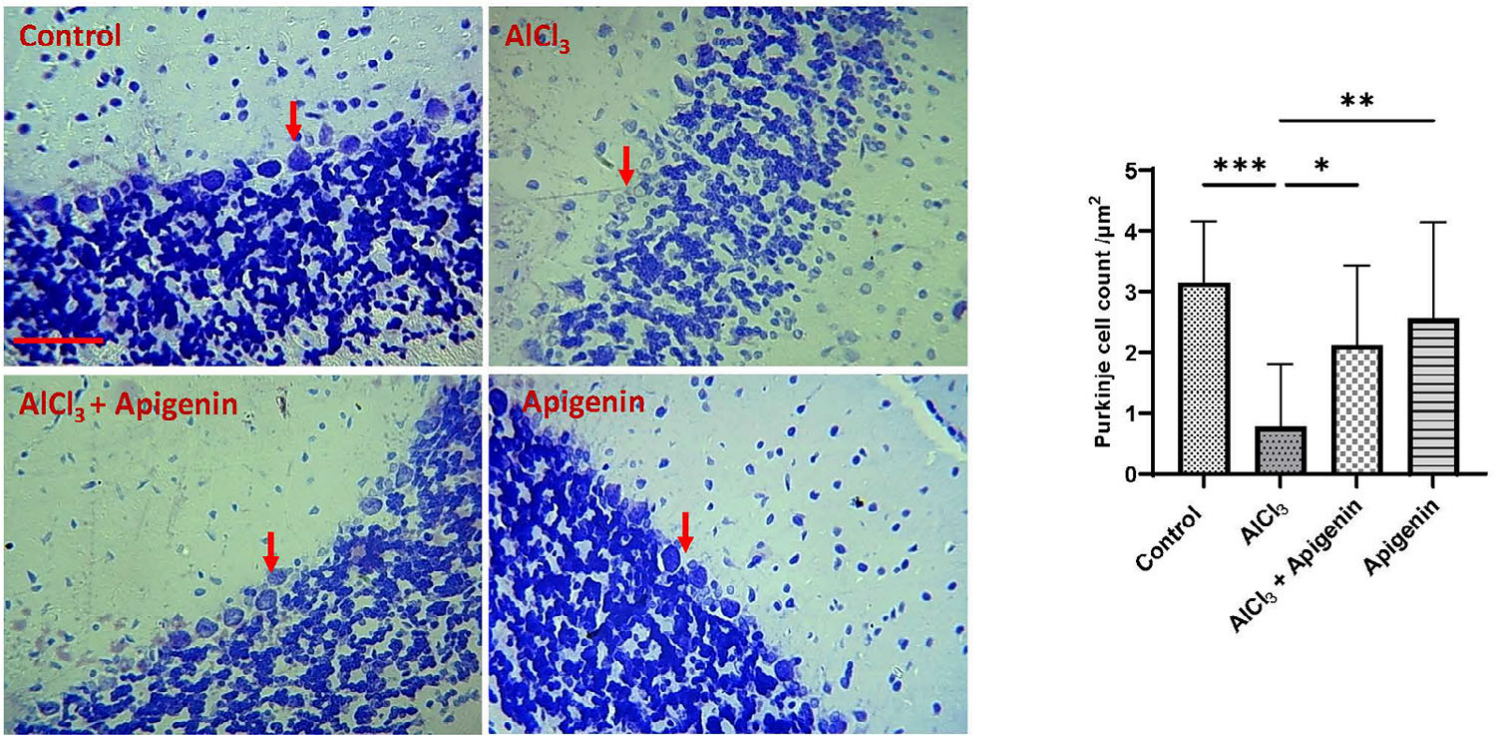
Cerebellum, cresyl violet stain. Note loss of Purkinje cell layer (red arrows) in AlCl_3_ group. These were rescued with concurrent administration of apigenin. Scale bar, 50 µm.

Histopathology revealed loss of the Purkinje cell layer in the AlCl_3_‐intoxicated rats (Figure 7). Amazingly, Purkinje cell layer restoration was observed in rats co‐treated with AlCl_3_ and apigenin and rats on apigenin alone relative to rats exposed to AlCl_3_ (Figure 7).

## 4 DISCUSSION

The study investigated antioxidant, neuromodulatory, and memory‐enhancing actions of apigenin in ameliorating AlCl_3_‐induced neurotoxicity in Wistar rats. The NORT is a straightforward exercise that takes advantage of the natural inclination of rats to investigate unfamiliar stimuli rather than ones they are familiar with.^52^ Specifically, it is one of the neurobehavioral tests for determining learning and memory outcomes in animal models of cognitive impairment. This study showed that apigenin was able to effectively mitigate and restore cognitive impairment associated with AlCl_3_‐induced neurotoxicity, as indicated by a reduction in the recognition index. In fact, apigenin treatment significantly improved learning and memory. In this study, administration of apigenin together with AlCl_3_ improved the recognition index, thereby mitigating neurobehavioral deficit and cognitive impairment associated with AlCl_3_‐induced neurotoxicity. Several studies have reported the induction of neurobehavioral deficit and cognitive impairment by AlCl_3_ toxicity.^53^, ^54^ Hence, the use of apigenin as a food supplement for the management of cognitive impairment could allow for a novel therapeutic regimen in neuroscience.

The results we obtained in this study showed that NO content increased significantly in rats that were exposed to only AlCl_3_. This is indicative of neuroinflammation and of neurotoxicity as enhanced by AlCl_3_ intoxication. However, treatment with apigenin significantly reduced NO content. This finding attests to the anti‐inflammatory and neuroprotective action of apigenin against lipopolysaccharide‐induced inflammation and inorganic arsenic salt‐induced toxicity in PC12 cells.^55^, ^56^ Toxicity associated with AlCl_3_ was further assessed using an AChE activity assay. In the present study, AChE activity was significantly higher following AlCl_3_ toxicity. Previous research findings documented the inhibition of AChE activity for the management of cognitive impairment and neurobehavioral deficit.^57^, ^58^ In our study, treatment of rats with apigenin caused significant inhibition of AChE activity. This also confirms the neuroprotective action of apigenin as previously reported by mitigating the abnormal accumulation of misfolded Aβ in AD.^59^, ^60^ Nitrosative stress and neuroinflammation could also trigger increased AChE activity, as observed in this study.^61^ Overall, apigenin mitigated neurotoxicity, neuroinflammation, and nitrosative stress via a reduction in the content of NO and AChE activity.

Biomarkers of oxidative stress following the induction of neurotoxicity by AlCl_3_ were assessed in brain tissues. We observed that AlCl_3_‐induced neurotoxicity precipitated oxidative stress with increased production of H_2_O_2_ and MDA. It is worth noting that the treatment of rats exposed to AlCl_3_ toxicity with apigenin ameliorated oxidative stress, as indicated by the significant reduction in H_2_O_2_ generation and MDA content. The results obtained are in conformity with recent documented results on the antioxidative action of apigenin.^62^, ^63^

Reactive oxygen species detoxification within cells depends on the GSH system.^64^ Lower levels of reduced glutathione (GSH) were recorded in the group administered AlCl3 only. The GSH is an intracellular antioxidant defense system.^65^ However, there was a significant increase in the content of GSH in rats treated with apigenin. The observable improvement in the content of GSH is an indication of the antioxidant and neuroprotective actions of apigenin. The depletion of the content of GSH has been reported to be a biomarker of oxidative injury in many disease conditions, including neurodegenerative diseases.^66^ Hence, the depletion of GSH content caused by AlCl_3_ is indicative of oxidative stress.^68^, ^69^

GST plays a crucial role in cellular defense by decreasing the toxicity of many hydrophobic and electrophilic intermediates, including drugs, carcinogens, and products of oxidative stress. It accomplishes this by catalyzing the conjugation of GSH with electrophilic intermediates.^70^ We recorded significant increases in the activity of GST in rats intoxicated with AlCl_3_. The observed increase in GST activity could be attributed to oxidative stress‐induced adaptive response, which has been documented elsewhere. However, we also observed significant improvement in GST activity in rats treated with apigenin. Furthermore, the activities of SOD and GPx were found to decrease significantly in rats exposed to only AlCl_3_. SOD is in the first line of defense against oxidative stress by dismutation of the superoxide anion radicals to hydrogen peroxide (H_2_O_2_) and oxygen (O_2_). Therefore, the inhibition of SOD activity by AlCl_3_ toxicity could have aggravated oxidative stress. A similar observation was recorded as AlCl_3_ toxicity inhibited GPx, although, this was not significant. Final detoxification of H_2_O_2_ and other hydroperoxides was performed by GPx. The impact of the toxicity was significant in this study, which might have been due to the duration of the experiment.

Histopathology revealed a loss of the Purkinje cell layer in AlCl_3_‐intoxicated rats. Several research findings have attributed loss of the Purkinje cell layer to neurodegeneration, neurobehavioral deficit, motor dysfunction, cognitive impairment, and memory loss.^71^, ^72^, ^73^, ^74^ We discovered that concurrent administration of apigenin rescued the cerebellum from Purkinje cell layer pathology, as indicated in the graphical abstract.

The results of this study demonstrate the effectiveness of apigenin as an antioxidant, anti‐inflammatory, and neuroprotective agent that effectively mitigated neurotoxicity, neuroinflammation, oxidative stress, and memory loss. Therefore, fruits and vegetables that contain apigenin could be employed for the management of cognitive impairment and memory loss associated with Alzheimer's disease.

## AUTHOR CONTRIBUTIONS

All authors contributed to the study conception and design. The authors, Ademola A. Oyagbemi, Omowumi M. Femi‐Akinlosotu, Adedunsola A. Obasa, and Moses S. Ojo, designed the experiment. Adeola T. Salami, Temitayo O. Ajibade, Charles E. Onukak, Olumayowa O. Igado, Oluwaseun O. Esan, Taiwo O. Oyagbemi, Adewunmi V. Adeogun, Omolola V. Awoyomi, and Joseph E. Ikokide performed laboratory work, biochemical assays, immunohistochemistry, and statistical analysis. Ademola A. Oyagbemi, Ishmael F. Jaja, Olufunke E. Ola‐Davies, Temidayo O. Omobowale, Adebowale B. Saba, Oluwafemi O. Oguntibeju, Evaristus Nwulia, and Momoh A. Yakubu supervised the experiment, prepared the manuscript, and proofread and approved the submission.

## CONFLICT OF INTEREST STATEMENT

The authors declare no conflicts of interest. Author disclosures are available in supporting information.

## CONSENT STATEMENT

We confirm that informed consent for human subjects was not necessary.

## Supporting information



Supporting Information

## Data Availability

The data that support the findings of this study are available from the corresponding author upon reasonable request.
