# Supplementary material for: Apigenin mitigates oxidative stress, neuroinflammation, and cognitive impairment but enhances learning and memory in aluminum chloride‐induced neurotoxicity in rats
Source: Alzheimers Dement. 2025 May 2;21(5):e70223. doi: 10.1002/alz.70223 (PMC12441593; doi:10.1002/alz.70223)
Supplement: Supplementary file 1 — Supporting Information [file ALZ-21-e70223-s001.pdf]

ICMJE DISCLOSURE FORM

Date: March 4, 2025

Your Name: Adewumi Victoria Adeogun

Manuscript Title: Apigenin mitigates oxidative stress, neuroinflammation, and cognitive impairment but enhances learning and memory in aluminum chloride-induced neurotoxicity in rats

Manuscript Number (if known): ADJ-D-24-02699R1

In the interest of transparency, we ask you to disclose all relationships/activities/interests listed below that are related to the content of your manuscript. "Related" means any relation with for-profit or not-for-profit third parties whose interests may be affected by the content of the manuscript. Disclosure represents a commitment to transparency and does not necessarily indicate a bias. If you are in doubt about whether to list a relationship/activity/interest, it is preferable that you do so.

The author's relationships/activities/interests should be defined broadly. For example, if your manuscript pertains to the epidemiology of hypertension, you should declare all relationships with manufacturers of antihypertensive medication, even if that medication is not mentioned in the manuscript.

In item #1 below, report all support for the work reported in this manuscript without time limit. For all other items, the time frame for disclosure is the past 36 months.

		Name all entities with whom you have this relationship or indicate none (add rows as needed)	Specifications/Comments (e.g., if payments were made to you or to your institution)
		**Time frame: Since the initial planning of the work**	
1	All support for the present manuscript (e.g., funding, provision of study materials, medical writing, article processing charges, etc.) **No time limit for this item.**	☐ None<br>None	None<br><br>Click the tab key to add additional rows.
		**Time frame: past 36 months**	
2	Grants or contracts from any entity (if not indicated in item #1 above).	☐ **None**<br>None	None
3	Royalties or licenses	☐ **None**<br>None	None

		Name all entities with whom you have this relationship or indicate none (add rows as needed)	Specifications/Comments (e.g., if payments were made to you or to your institution)
**4**	Consulting fees	☐ **None**	
		None	None
**5**	Payment or honoraria for lectures, presentations, speakers bureaus, manuscript writing or educational events	☐ **None**	
		None	None
**6**	Payment for expert testimony	☐ **None**	
		None	None
**7**	Support for attending meetings and/or travel	☐ **None**	
		None	None
**8**	Patents planned, issued or pending	☐ **None**	
		None	None
**9**	Participation on a Data Safety Monitoring Board or Advisory Board	☐ **None**	
		None	None
**10**	Leadership or fiduciary role in other board, society, committee or advocacy group, paid or unpaid	☐ **None**	
		None	None

		Name all entities with whom you have this relationship or indicate none (add rows as needed)	Specifications/Comments (e.g., if payments were made to you or to your institution)
**11**	Stock or stock options	☐ **None**	
		None	None
**12**	Receipt of equipment, materials, drugs, medical writing, gifts or other services	☐ **None**	
		None	None
**13**	Other financial or non-financial interests	☐ **None**	
		None	None

Please place an "X" next to the following statement to indicate your agreement:

X I certify that I have answered every question and have not altered the wording of any of the questions on this form.

ICMJE DISCLOSURE FORM

Date: March 4, 2025

Your Name: Temitayo Olabisi Ajibade

Manuscript Title: Apigenin mitigates oxidative stress, neuroinflammation, and cognitive impairment but enhances learning and memory in aluminum chloride-induced neurotoxicity in rats

Manuscript Number (if known): ADJ-D-24-02699R1

In the interest of transparency, we ask you to disclose all relationships/activities/interests listed below that are related to the content of your manuscript. "Related" means any relation with for-profit or not-for-profit third parties whose interests may be affected by the content of the manuscript. Disclosure represents a commitment to transparency and does not necessarily indicate a bias. If you are in doubt about whether to list a relationship/activity/interest, it is preferable that you do so.

The author's relationships/activities/interests should be defined broadly. For example, if your manuscript pertains to the epidemiology of hypertension, you should declare all relationships with manufacturers of antihypertensive medication, even if that medication is not mentioned in the manuscript.

In item #1 below, report all support for the work reported in this manuscript without time limit. For all other items, the time frame for disclosure is the past 36 months.

		**Name all entities with whom you have this relationship or indicate none (add rows as needed)**	**Specifications/Comments (e.g., if payments were made to you or to your institution)**
**Time frame: Since the initial planning of the work**			
1	All support for the present manuscript (e.g., funding, provision of study materials, medical writing, article processing charges, etc.) **No time limit for this item.**	☐ None	
		None	None
			Click the tab key to add additional rows.
**Time frame: past 36 months**			
**2**	Grants or contracts from any entity (if not indicated in item #1 above).	☐ **None**	
		None	None
**3**	Royalties or licenses	☐ **None**	
		None	None

		Name all entities with whom you have this relationship or indicate none (add rows as needed)	Specifications/Comments (e.g., if payments were made to you or to your institution)
**4**	Consulting fees	☐ **None**	
		None	None
**5**	Payment or honoraria for lectures, presentations, speakers bureaus, manuscript writing or educational events	☐ **None**	
		None	None
**6**	Payment for expert testimony	☐ **None**	
		None	None
**7**	Support for attending meetings and/or travel	☐ **None**	
		None	None
**8**	Patents planned, issued or pending	☐ **None**	
		None	None
**9**	Participation on a Data Safety Monitoring Board or Advisory Board	☐ **None**	
		None	None
**10**	Leadership or fiduciary role in other board, society, committee or advocacy group, paid or unpaid	☐ **None**	
		None	None

		Name all entities with whom you have this relationship or indicate none (add rows as needed)	Specifications/Comments (e.g., if payments were made to you or to your institution)
**11**	Stock or stock options	☐ **None**	
		None	None
**12**	Receipt of equipment, materials, drugs, medical writing, gifts or other services	☐ **None**	
		None	None
**13**	Other financial or non-financial interests	☐ **None**	
		None	None

Please place an "X" next to the following statement to indicate your agreement:

X I certify that I have answered every question and have not altered the wording of any of the questions on this form.

ICMJE DISCLOSURE FORM

Date: March 4, 2025

Your Name: Omolola Victoria Awoyomi

Manuscript Title: Apigenin mitigates oxidative stress, neuroinflammation, and cognitive impairment but enhances learning and memory in aluminum chloride-induced neurotoxicity in rats

Manuscript Number (if known): ADJ-D-24-02699R1

In the interest of transparency, we ask you to disclose all relationships/activities/interests listed below that are related to the content of your manuscript. "Related" means any relation with for-profit or not-for-profit third parties whose interests may be affected by the content of the manuscript. Disclosure represents a commitment to transparency and does not necessarily indicate a bias. If you are in doubt about whether to list a relationship/activity/interest, it is preferable that you do so.

The author's relationships/activities/interests should be defined broadly. For example, if your manuscript pertains to the epidemiology of hypertension, you should declare all relationships with manufacturers of antihypertensive medication, even if that medication is not mentioned in the manuscript.

In item #1 below, report all support for the work reported in this manuscript without time limit. For all other items, the time frame for disclosure is the past 36 months.

		Name all entities with whom you have this relationship or indicate none (add rows as needed)	Specifications/Comments (e.g., if payments were made to you or to your institution)
**Time frame: Since the initial planning of the work**			
1	All support for the present manuscript (e.g., funding, provision of study materials, medical writing, article processing charges, etc.) **No time limit for this item.**	☐ None<br>None	None<br><br>Click the tab key to add additional rows.
**Time frame: past 36 months**			
**2**	Grants or contracts from any entity (if not indicated in item #1 above).	☐ **None**<br>None	None
**3**	Royalties or licenses	☐ **None**<br>None	None

		Name all entities with whom you have this relationship or indicate none (add rows as needed)	Specifications/Comments (e.g., if payments were made to you or to your institution)
**4**	Consulting fees	☐ **None**	
		None	None
**5**	Payment or honoraria for lectures, presentations, speakers bureaus, manuscript writing or educational events	☐ **None**	
		None	None
**6**	Payment for expert testimony	☐ **None**	
		None	None
**7**	Support for attending meetings and/or travel	☐ **None**	
		None	None
**8**	Patents planned, issued or pending	☐ **None**	
		None	None
**9**	Participation on a Data Safety Monitoring Board or Advisory Board	☐ **None**	
		None	None
**10**	Leadership or fiduciary role in other board, society, committee or advocacy group, paid or unpaid	☐ **None**	
		None	None

		Name all entities with whom you have this relationship or indicate none (add rows as needed)	Specifications/Comments (e.g., if payments were made to you or to your institution)
**11**	Stock or stock options	☐ **None**	
		None	None
**12**	Receipt of equipment, materials, drugs, medical writing, gifts or other services	☐ **None**	
		None	None
**13**	Other financial or non-financial interests	☐ **None**	
		None	None

Please place an "X" next to the following statement to indicate your agreement:

X I certify that I have answered every question and have not altered the wording of any of the questions on this form.

ICMJE DISCLOSURE FORM

Date: March 4, 2025

Your Name: Oluwaseun Olarenwaju Esan

Manuscript Title: Apigenin mitigates oxidative stress, neuroinflammation, and cognitive impairment but enhances learning and memory in aluminum chloride-induced neurotoxicity in rats

Manuscript Number (if known): ADJ-D-24-02699R1

In the interest of transparency, we ask you to disclose all relationships/activities/interests listed below that are related to the content of your manuscript. "Related" means any relation with for-profit or not-for-profit third parties whose interests may be affected by the content of the manuscript. Disclosure represents a commitment to transparency and does not necessarily indicate a bias. If you are in doubt about whether to list a relationship/activity/interest, it is preferable that you do so.

The author's relationships/activities/interests should be defined broadly. For example, if your manuscript pertains to the epidemiology of hypertension, you should declare all relationships with manufacturers of antihypertensive medication, even if that medication is not mentioned in the manuscript.

In item #1 below, report all support for the work reported in this manuscript without time limit. For all other items, the time frame for disclosure is the past 36 months.

		**Name all entities with whom you have this relationship or indicate none (add rows as needed)**	**Specifications/Comments (e.g., if payments were made to you or to your institution)**
**Time frame: Since the initial planning of the work**			
1	All support for the present manuscript (e.g., funding, provision of study materials, medical writing, article processing charges, etc.) **No time limit for this item.**	☐ None<br>None	None<br><br>Click the tab key to add additional rows.
**Time frame: past 36 months**			
**2**	Grants or contracts from any entity (if not indicated in item #1 above).	☐ **None**<br>None	None
**3**	Royalties or licenses	☐ **None**<br>None	None

		Name all entities with whom you have this relationship or indicate none (add rows as needed)	Specifications/Comments (e.g., if payments were made to you or to your institution)
**4**	Consulting fees	☐ **None**	
		None	None
**5**	Payment or honoraria for lectures, presentations, speakers bureaus, manuscript writing or educational events	☐ **None**	
		None	None
**6**	Payment for expert testimony	☐ **None**	
		None	None
**7**	Support for attending meetings and/or travel	☐ **None**	
		None	None
**8**	Patents planned, issued or pending	☐ **None**	
		None	None
**9**	Participation on a Data Safety Monitoring Board or Advisory Board	☐ **None**	
		None	None
**10**	Leadership or fiduciary role in other board, society, committee or advocacy group, paid or unpaid	☐ **None**	
		None	None

		Name all entities with whom you have this relationship or indicate none (add rows as needed)	Specifications/Comments (e.g., if payments were made to you or to your institution)
**11**	Stock or stock options	☐ **None**	
		None	None
**12**	Receipt of equipment, materials, drugs, medical writing, gifts or other services	☐ **None**	
		None	None
**13**	Other financial or non-financial interests	☐ **None**	
		None	None

Please place an "X" next to the following statement to indicate your agreement:

X I certify that I have answered every question and have not altered the wording of any of the questions on this form.

ICMJE DISCLOSURE FORM

Date: March 4, 2025

Your Name: Omowumi Moromoke Femi-Akinlosotu

Manuscript Title: Apigenin mitigates oxidative stress, neuroinflammation, and cognitive impairment but enhances learning and memory in aluminum chloride-induced neurotoxicity in rats

Manuscript Number (if known): ADJ-D-24-02699R1

In the interest of transparency, we ask you to disclose all relationships/activities/interests listed below that are related to the content of your manuscript. "Related" means any relation with for-profit or not-for-profit third parties whose interests may be affected by the content of the manuscript. Disclosure represents a commitment to transparency and does not necessarily indicate a bias. If you are in doubt about whether to list a relationship/activity/interest, it is preferable that you do so.

The author's relationships/activities/interests should be defined broadly. For example, if your manuscript pertains to the epidemiology of hypertension, you should declare all relationships with manufacturers of antihypertensive medication, even if that medication is not mentioned in the manuscript.

In item #1 below, report all support for the work reported in this manuscript without time limit. For all other items, the time frame for disclosure is the past 36 months.

		Name all entities with whom you have this relationship or indicate none (add rows as needed)	Specifications/Comments (e.g., if payments were made to you or to your institution)
**Time frame: Since the initial planning of the work**			
1	All support for the present manuscript (e.g., funding, provision of study materials, medical writing, article processing charges, etc.) **No time limit for this item.**	☐ None<br>None	None
			Click the tab key to add additional rows.
**Time frame: past 36 months**			
**2**	Grants or contracts from any entity (if not indicated in item #1 above).	☐ **None**<br>None	None
**3**	Royalties or licenses	☐ **None**<br>None	None

		Name all entities with whom you have this relationship or indicate none (add rows as needed)	Specifications/Comments (e.g., if payments were made to you or to your institution)
**4**	Consulting fees	☐ **None**	
		None	None
**5**	Payment or honoraria for lectures, presentations, speakers bureaus, manuscript writing or educational events	☐ **None**	
		None	None
**6**	Payment for expert testimony	☐ **None**	
		None	None
**7**	Support for attending meetings and/or travel	☐ **None**	
		None	None
**8**	Patents planned, issued or pending	☐ **None**	
		None	None
**9**	Participation on a Data Safety Monitoring Board or Advisory Board	☐ **None**	
		None	None
**10**	Leadership or fiduciary role in other board, society, committee or advocacy group, paid or unpaid	☐ **None**	
		None	None

		Name all entities with whom you have this relationship or indicate none (add rows as needed)	Specifications/Comments (e.g., if payments were made to you or to your institution)
**11**	Stock or stock options	☐ **None**	
		None	None
**12**	Receipt of equipment, materials, drugs, medical writing, gifts or other services	☐ **None**	
		None	None
**13**	Other financial or non-financial interests	☐ **None**	
		None	None

Please place an "X" next to the following statement to indicate your agreement:

X I certify that I have answered every question and have not altered the wording of any of the questions on this form.

ICMJE DISCLOSURE FORM

Date: March 4, 2025

Your Name: Olumayowa Olawumi Igado

Manuscript Title: Apigenin mitigates oxidative stress, neuroinflammation, and cognitive impairment but enhances learning and memory in aluminum chloride-induced neurotoxicity in rats

Manuscript Number (if known): ADJ-D-24-02699R1

In the interest of transparency, we ask you to disclose all relationships/activities/interests listed below that are related to the content of your manuscript. "Related" means any relation with for-profit or not-for-profit third parties whose interests may be affected by the content of the manuscript. Disclosure represents a commitment to transparency and does not necessarily indicate a bias. If you are in doubt about whether to list a relationship/activity/interest, it is preferable that you do so.

The author's relationships/activities/interests should be defined broadly. For example, if your manuscript pertains to the epidemiology of hypertension, you should declare all relationships with manufacturers of antihypertensive medication, even if that medication is not mentioned in the manuscript.

In item #1 below, report all support for the work reported in this manuscript without time limit. For all other items, the time frame for disclosure is the past 36 months.

		**Name all entities with whom you have this relationship or indicate none (add rows as needed)**	**Specifications/Comments (e.g., if payments were made to you or to your institution)**
**Time frame: Since the initial planning of the work**			
1	All support for the present manuscript (e.g., funding, provision of study materials, medical writing, article processing charges, etc.) **No time limit for this item.**	☐ None	
		None	None
			Click the tab key to add additional rows.
**Time frame: past 36 months**			
**2**	Grants or contracts from any entity (if not indicated in item #1 above).	☐ **None**	
		None	None
**3**	Royalties or licenses	☐ **None**	
		None	None

		Name all entities with whom you have this relationship or indicate none (add rows as needed)	Specifications/Comments (e.g., if payments were made to you or to your institution)
**4**	Consulting fees	☐ **None**	
		None	None
**5**	Payment or honoraria for lectures, presentations, speakers bureaus, manuscript writing or educational events	☐ **None**	
		None	None
**6**	Payment for expert testimony	☐ **None**	
		None	None
**7**	Support for attending meetings and/or travel	☐ **None**	
		None	None
**8**	Patents planned, issued or pending	☐ **None**	
		None	None
**9**	Participation on a Data Safety Monitoring Board or Advisory Board	☐ **None**	
		None	None
**10**	Leadership or fiduciary role in other board, society, committee or advocacy group, paid or unpaid	☐ **None**	
		None	None

		Name all entities with whom you have this relationship or indicate none (add rows as needed)	Specifications/Comments (e.g., if payments were made to you or to your institution)
**11**	Stock or stock options	☐ **None**	
		None	None
**12**	Receipt of equipment, materials, drugs, medical writing, gifts or other services	☐ **None**	
		None	None
**13**	Other financial or non-financial interests	☐ **None**	
		None	None

Please place an "X" next to the following statement to indicate your agreement:

X I certify that I have answered every question and have not altered the wording of any of the questions on this form.

ICMJE DISCLOSURE FORM

Date: March 4, 2025

Your Name: Joseph E Ikokide

Manuscript Title: Apigenin mitigates oxidative stress, neuroinflammation, and cognitive impairment but enhances learning and memory in aluminum chloride-induced neurotoxicity in rats

Manuscript Number (if known): ADJ-D-24-02699R1

In the interest of transparency, we ask you to disclose all relationships/activities/interests listed below that are related to the content of your manuscript. "Related" means any relation with for-profit or not-for-profit third parties whose interests may be affected by the content of the manuscript. Disclosure represents a commitment to transparency and does not necessarily indicate a bias. If you are in doubt about whether to list a relationship/activity/interest, it is preferable that you do so.

The author's relationships/activities/interests should be defined broadly. For example, if your manuscript pertains to the epidemiology of hypertension, you should declare all relationships with manufacturers of antihypertensive medication, even if that medication is not mentioned in the manuscript.

In item #1 below, report all support for the work reported in this manuscript without time limit. For all other items, the time frame for disclosure is the past 36 months.

		Name all entities with whom you have this relationship or indicate none (add rows as needed)	Specifications/Comments (e.g., if payments were made to you or to your institution)
Time frame: Since the initial planning of the work			
1	All support for the present manuscript (e.g., funding, provision of study materials, medical writing, article processing charges, etc.) **No time limit for this item.**	☐ None<br>None	None
			Click the tab key to add additional rows.
Time frame: past 36 months			
2	Grants or contracts from any entity (if not indicated in item #1 above).	☐ **None**<br>None	None
3	Royalties or licenses	☐ **None**<br>None	None

		Name all entities with whom you have this relationship or indicate none (add rows as needed)	Specifications/Comments (e.g., if payments were made to you or to your institution)
**4**	Consulting fees	☐ **None**	
		None	None
**5**	Payment or honoraria for lectures, presentations, speakers bureaus, manuscript writing or educational events	☐ **None**	
		None	None
**6**	Payment for expert testimony	☐ **None**	
		None	None
**7**	Support for attending meetings and/or travel	☐ **None**	
		None	None
**8**	Patents planned, issued or pending	☐ **None**	
		None	None
**9**	Participation on a Data Safety Monitoring Board or Advisory Board	☐ **None**	
		None	None
**10**	Leadership or fiduciary role in other board, society, committee or advocacy group, paid or unpaid	☐ **None**	
		None	None

		Name all entities with whom you have this relationship or indicate none (add rows as needed)	Specifications/Comments (e.g., if payments were made to you or to your institution)
**11**	Stock or stock options	☐ **None**	
		None	None
**12**	Receipt of equipment, materials, drugs, medical writing, gifts or other services	☐ **None**	
		None	None
**13**	Other financial or non-financial interests	☐ **None**	
		None	None

Please place an "X" next to the following statement to indicate your agreement:

X I certify that I have answered every question and have not altered the wording of any of the questions on this form.

ICMJE DISCLOSURE FORM

Date: March 4, 2025

Your Name: Ishmael Festus Jaja

Manuscript Title: Apigenin mitigates oxidative stress, neuroinflammation, and cognitive impairment but enhances learning and memory in aluminum chloride-induced neurotoxicity in rats

Manuscript Number (if known): ADJ-D-24-02699R1

In the interest of transparency, we ask you to disclose all relationships/activities/interests listed below that are related to the content of your manuscript. "Related" means any relation with for-profit or not-for-profit third parties whose interests may be affected by the content of the manuscript. Disclosure represents a commitment to transparency and does not necessarily indicate a bias. If you are in doubt about whether to list a relationship/activity/interest, it is preferable that you do so.

The author's relationships/activities/interests should be defined broadly. For example, if your manuscript pertains to the epidemiology of hypertension, you should declare all relationships with manufacturers of antihypertensive medication, even if that medication is not mentioned in the manuscript.

In item #1 below, report all support for the work reported in this manuscript without time limit. For all other items, the time frame for disclosure is the past 36 months.

		**Name all entities with whom you have this relationship or indicate none (add rows as needed)**	**Specifications/Comments (e.g., if payments were made to you or to your institution)**
**Time frame: Since the initial planning of the work**			
1	All support for the present manuscript (e.g., funding, provision of study materials, medical writing, article processing charges, etc.) **No time limit for this item.**	☐ None	
		None	None
			Click the tab key to add additional rows.
**Time frame: past 36 months**			
**2**	Grants or contracts from any entity (if not indicated in item #1 above).	☐ **None**	
		None	None
**3**	Royalties or licenses	☐ **None**	
		None	None

		Name all entities with whom you have this relationship or indicate none (add rows as needed)	Specifications/Comments (e.g., if payments were made to you or to your institution)
**4**	Consulting fees	☐ **None**	
		None	None
**5**	Payment or honoraria for lectures, presentations, speakers bureaus, manuscript writing or educational events	☐ **None**	
		None	None
**6**	Payment for expert testimony	☐ **None**	
		None	None
**7**	Support for attending meetings and/or travel	☐ **None**	
		None	None
**8**	Patents planned, issued or pending	☐ **None**	
		None	None
**9**	Participation on a Data Safety Monitoring Board or Advisory Board	☐ **None**	
		None	None
**10**	Leadership or fiduciary role in other board, society, committee or advocacy group, paid or unpaid	☐ **None**	
		None	None

		Name all entities with whom you have this relationship or indicate none (add rows as needed)	Specifications/Comments (e.g., if payments were made to you or to your institution)
**11**	Stock or stock options	☐ **None**	
		None	None
**12**	Receipt of equipment, materials, drugs, medical writing, gifts or other services	☐ **None**	
		None	None
**13**	Other financial or non-financial interests	☐ **None**	
		None	None

Please place an "X" next to the following statement to indicate your agreement:

X I certify that I have answered every question and have not altered the wording of any of the questions on this form.

ICMJE DISCLOSURE FORM

Date: March 4, 2025

Your Name: Evaristus Nwulia

Manuscript Title: Apigenin mitigates oxidative stress, neuroinflammation, and cognitive impairment but enhances learning and memory in aluminum chloride-induced neurotoxicity in rats

Manuscript Number (if known): ADJ-D-24-02699R1

In the interest of transparency, we ask you to disclose all relationships/activities/interests listed below that are related to the content of your manuscript. "Related" means any relation with for-profit or not-for-profit third parties whose interests may be affected by the content of the manuscript. Disclosure represents a commitment to transparency and does not necessarily indicate a bias. If you are in doubt about whether to list a relationship/activity/interest, it is preferable that you do so.

The author's relationships/activities/interests should be defined broadly. For example, if your manuscript pertains to the epidemiology of hypertension, you should declare all relationships with manufacturers of antihypertensive medication, even if that medication is not mentioned in the manuscript.

In item #1 below, report all support for the work reported in this manuscript without time limit. For all other items, the time frame for disclosure is the past 36 months.

		Name all entities with whom you have this relationship or indicate none (add rows as needed)	Specifications/Comments (e.g., if payments were made to you or to your institution)
**Time frame: Since the initial planning of the work**			
1	All support for the present manuscript (e.g., funding, provision of study materials, medical writing, article processing charges, etc.) **No time limit for this item.**	☐ None<br>None	None<br><br>Click the tab key to add additional rows.
**Time frame: past 36 months**			
**2**	Grants or contracts from any entity (if not indicated in item #1 above).	☐ **None**<br>None	None
**3**	Royalties or licenses	☐ **None**<br>None	None

		Name all entities with whom you have this relationship or indicate none (add rows as needed)	Specifications/Comments (e.g., if payments were made to you or to your institution)
**4**	Consulting fees	☐ **None**	
		None	None
**5**	Payment or honoraria for lectures, presentations, speakers bureaus, manuscript writing or educational events	☐ **None**	
		None	None
**6**	Payment for expert testimony	☐ **None**	
		None	None
**7**	Support for attending meetings and/or travel	☐ **None**	
		None	None
**8**	Patents planned, issued or pending	☐ **None**	
		None	None
**9**	Participation on a Data Safety Monitoring Board or Advisory Board	☐ **None**	
		None	None
**10**	Leadership or fiduciary role in other board, society, committee or advocacy group, paid or unpaid	☐ **None**	
		None	None

		Name all entities with whom you have this relationship or indicate none (add rows as needed)	Specifications/Comments (e.g., if payments were made to you or to your institution)
**11**	Stock or stock options	☐ **None**	
		None	None
**12**	Receipt of equipment, materials, drugs, medical writing, gifts or other services	☐ **None**	
		None	None
**13**	Other financial or non-financial interests	☐ **None**	
		None	None

Please place an "X" next to the following statement to indicate your agreement:

X I certify that I have answered every question and have not altered the wording of any of the questions on this form.

ICMJE DISCLOSURE FORM

Date: March 4, 2025

Your Name: Adedunsola Adewunmi Obasa

Manuscript Title: Apigenin mitigates oxidative stress, neuroinflammation, and cognitive impairment but enhances learning and memory in aluminum chloride-induced neurotoxicity in rats

Manuscript Number (if known): ADJ-D-24-02699R1

In the interest of transparency, we ask you to disclose all relationships/activities/interests listed below that are related to the content of your manuscript. "Related" means any relation with for-profit or not-for-profit third parties whose interests may be affected by the content of the manuscript. Disclosure represents a commitment to transparency and does not necessarily indicate a bias. If you are in doubt about whether to list a relationship/activity/interest, it is preferable that you do so.

The author's relationships/activities/interests should be defined broadly. For example, if your manuscript pertains to the epidemiology of hypertension, you should declare all relationships with manufacturers of antihypertensive medication, even if that medication is not mentioned in the manuscript.

In item #1 below, report all support for the work reported in this manuscript without time limit. For all other items, the time frame for disclosure is the past 36 months.

		Name all entities with whom you have this relationship or indicate none (add rows as needed)	Specifications/Comments (e.g., if payments were made to you or to your institution)
**Time frame: Since the initial planning of the work**			
1	All support for the present manuscript (e.g., funding, provision of study materials, medical writing, article processing charges, etc.) **No time limit for this item.**	☐ None<br>None	None
			Click the tab key to add additional rows.
**Time frame: past 36 months**			
**2**	Grants or contracts from any entity (if not indicated in item #1 above).	☐ **None**<br>None	None
**3**	Royalties or licenses	☐ **None**<br>None	None

		Name all entities with whom you have this relationship or indicate none (add rows as needed)	Specifications/Comments (e.g., if payments were made to you or to your institution)
**4**	Consulting fees	☐ **None**	
		None	None
**5**	Payment or honoraria for lectures, presentations, speakers bureaus, manuscript writing or educational events	☐ **None**	
		None	None
**6**	Payment for expert testimony	☐ **None**	
		None	None
**7**	Support for attending meetings and/or travel	☐ **None**	
		None	None
**8**	Patents planned, issued or pending	☐ **None**	
		None	None
**9**	Participation on a Data Safety Monitoring Board or Advisory Board	☐ **None**	
		None	None
**10**	Leadership or fiduciary role in other board, society, committee or advocacy group, paid or unpaid	☐ **None**	
		None	None

		Name all entities with whom you have this relationship or indicate none (add rows as needed)	Specifications/Comments (e.g., if payments were made to you or to your institution)
**11**	Stock or stock options	☐ **None**	
		None	None
**12**	Receipt of equipment, materials, drugs, medical writing, gifts or other services	☐ **None**	
		None	None
**13**	Other financial or non-financial interests	☐ **None**	
		None	None

Please place an "X" next to the following statement to indicate your agreement:

X I certify that I have answered every question and have not altered the wording of any of the questions on this form.

ICMJE DISCLOSURE FORM

Date: March 4, 2025

Your Name: Oluwafemi Omoniyi Oguntibeju

Manuscript Title: Apigenin mitigates oxidative stress, neuroinflammation, and cognitive impairment but enhances learning and memory in aluminum chloride-induced neurotoxicity in rats

Manuscript Number (if known): ADJ-D-24-02699R1

In the interest of transparency, we ask you to disclose all relationships/activities/interests listed below that are related to the content of your manuscript. "Related" means any relation with for-profit or not-for-profit third parties whose interests may be affected by the content of the manuscript. Disclosure represents a commitment to transparency and does not necessarily indicate a bias. If you are in doubt about whether to list a relationship/activity/interest, it is preferable that you do so.

The author's relationships/activities/interests should be defined broadly. For example, if your manuscript pertains to the epidemiology of hypertension, you should declare all relationships with manufacturers of antihypertensive medication, even if that medication is not mentioned in the manuscript.

In item #1 below, report all support for the work reported in this manuscript without time limit. For all other items, the time frame for disclosure is the past 36 months.

		**Name all entities with whom you have this relationship or indicate none (add rows as needed)**	**Specifications/Comments (e.g., if payments were made to you or to your institution)**
**Time frame: Since the initial planning of the work**			
1	All support for the present manuscript (e.g., funding, provision of study materials, medical writing, article processing charges, etc.) **No time limit for this item.**	☐ None<br>None	None<br>Click the tab key to add additional rows.
**Time frame: past 36 months**			
**2**	Grants or contracts from any entity (if not indicated in item #1 above).	☐ **None**<br>None	None
**3**	Royalties or licenses	☐ **None**<br>None	None

		Name all entities with whom you have this relationship or indicate none (add rows as needed)	Specifications/Comments (e.g., if payments were made to you or to your institution)
**4**	Consulting fees	☐ **None**	
		None	None
**5**	Payment or honoraria for lectures, presentations, speakers bureaus, manuscript writing or educational events	☐ **None**	
		None	None
**6**	Payment for expert testimony	☐ **None**	
		None	None
**7**	Support for attending meetings and/or travel	☐ **None**	
		None	None
**8**	Patents planned, issued or pending	☐ **None**	
		None	None
**9**	Participation on a Data Safety Monitoring Board or Advisory Board	☐ **None**	
		None	None
**10**	Leadership or fiduciary role in other board, society, committee or advocacy group, paid or unpaid	☐ **None**	
		None	None

		Name all entities with whom you have this relationship or indicate none (add rows as needed)	Specifications/Comments (e.g., if payments were made to you or to your institution)
**11**	Stock or stock options	☐ **None**	
		None	None
**12**	Receipt of equipment, materials, drugs, medical writing, gifts or other services	☐ **None**	
		None	None
**13**	Other financial or non-financial interests	☐ **None**	
		None	None

Please place an "X" next to the following statement to indicate your agreement:

X I certify that I have answered every question and have not altered the wording of any of the questions on this form.

ICMJE DISCLOSURE FORM

Date: March 4, 2025

Your Name: Moses Semilore Ojo

Manuscript Title: Apigenin mitigates oxidative stress, neuroinflammation, and cognitive impairment but enhances learning and memory in aluminum chloride-induced neurotoxicity in rats

Manuscript Number (if known): ADJ-D-24-02699R1

In the interest of transparency, we ask you to disclose all relationships/activities/interests listed below that are related to the content of your manuscript. "Related" means any relation with for-profit or not-for-profit third parties whose interests may be affected by the content of the manuscript. Disclosure represents a commitment to transparency and does not necessarily indicate a bias. If you are in doubt about whether to list a relationship/activity/interest, it is preferable that you do so.

The author's relationships/activities/interests should be defined broadly. For example, if your manuscript pertains to the epidemiology of hypertension, you should declare all relationships with manufacturers of antihypertensive medication, even if that medication is not mentioned in the manuscript.

In item #1 below, report all support for the work reported in this manuscript without time limit. For all other items, the time frame for disclosure is the past 36 months.

		**Name all entities with whom you have this relationship or indicate none (add rows as needed)**	**Specifications/Comments (e.g., if payments were made to you or to your institution)**
**Time frame: Since the initial planning of the work**			
1	All support for the present manuscript (e.g., funding, provision of study materials, medical writing, article processing charges, etc.) **No time limit for this item.**	☐ None<br>None	None
			Click the tab key to add additional rows.
**Time frame: past 36 months**			
**2**	Grants or contracts from any entity (if not indicated in item #1 above).	☐ **None**<br>None	None
**3**	Royalties or licenses	☐ **None**<br>None	None

		Name all entities with whom you have this relationship or indicate none (add rows as needed)	Specifications/Comments (e.g., if payments were made to you or to your institution)
**4**	Consulting fees	☐ **None**	
		None	None
**5**	Payment or honoraria for lectures, presentations, speakers bureaus, manuscript writing or educational events	☐ **None**	
		None	None
**6**	Payment for expert testimony	☐ **None**	
		None	None
**7**	Support for attending meetings and/or travel	☐ **None**	
		None	None
**8**	Patents planned, issued or pending	☐ **None**	
		None	None
**9**	Participation on a Data Safety Monitoring Board or Advisory Board	☐ **None**	
		None	None
**10**	Leadership or fiduciary role in other board, society, committee or advocacy group, paid or unpaid	☐ **None**	
		None	None

		Name all entities with whom you have this relationship or indicate none (add rows as needed)	Specifications/Comments (e.g., if payments were made to you or to your institution)
**11**	Stock or stock options	☐ **None**	
		None	None
**12**	Receipt of equipment, materials, drugs, medical writing, gifts or other services	☐ **None**	
		None	None
**13**	Other financial or non-financial interests	☐ **None**	
		None	None

Please place an "X" next to the following statement to indicate your agreement:

X I certify that I have answered every question and have not altered the wording of any of the questions on this form.

ICMJE DISCLOSURE FORM

**Date:**	March 4, 2025
**Your Name:**	Olufunke Eunice Ola-Davies
**Manuscript Title:**	Apigenin mitigates oxidative stress, neuroinflammation, and cognitive impairment but enhances learning and memory in aluminum chloride-induced neurotoxicity in rats
**Manuscript Number (if known):**	ADJ-D-24-02699R1

In the interest of transparency, we ask you to disclose all relationships/activities/interests listed below that are related to the content of your manuscript. "Related" means any relation with for-profit or not-for-profit third parties whose interests may be affected by the content of the manuscript. Disclosure represents a commitment to transparency and does not necessarily indicate a bias. If you are in doubt about whether to list a relationship/activity/interest, it is preferable that you do so.

The author's relationships/activities/interests should be defined broadly. For example, if your manuscript pertains to the epidemiology of hypertension, you should declare all relationships with manufacturers of antihypertensive medication, even if that medication is not mentioned in the manuscript.

In item #1 below, report all support for the work reported in this manuscript without time limit. For all other items, the time frame for disclosure is the past 36 months.

		**Name all entities with whom you have this relationship or indicate none (add rows as needed)**	**Specifications/Comments (e.g., if payments were made to you or to your institution)**
**Time frame: Since the initial planning of the work**			
1	All support for the present manuscript (e.g., funding, provision of study materials, medical writing, article processing charges, etc.) **No time limit for this item.**	☐ None	
		None	None
			Click the tab key to add additional rows.
**Time frame: past 36 months**			
**2**	Grants or contracts from any entity (if not indicated in item #1 above).	☐ **None**	
		None	None
**3**	Royalties or licenses	☐ **None**	
		None	None

		Name all entities with whom you have this relationship or indicate none (add rows as needed)	Specifications/Comments (e.g., if payments were made to you or to your institution)
**4**	Consulting fees	☐ **None**	
		None	None
**5**	Payment or honoraria for lectures, presentations, speakers bureaus, manuscript writing or educational events	☐ **None**	
		None	None
**6**	Payment for expert testimony	☐ **None**	
		None	None
**7**	Support for attending meetings and/or travel	☐ **None**	
		None	None
**8**	Patents planned, issued or pending	☐ **None**	
		None	None
**9**	Participation on a Data Safety Monitoring Board or Advisory Board	☐ **None**	
		None	None
**10**	Leadership or fiduciary role in other board, society, committee or advocacy group, paid or unpaid	☐ **None**	
		None	None

		Name all entities with whom you have this relationship or indicate none (add rows as needed)	Specifications/Comments (e.g., if payments were made to you or to your institution)
**11**	Stock or stock options	☐ **None**	
		None	None
**12**	Receipt of equipment, materials, drugs, medical writing, gifts or other services	☐ **None**	
		None	None
**13**	Other financial or non-financial interests	☐ **None**	
		None	None

Please place an "X" next to the following statement to indicate your agreement:

X I certify that I have answered every question and have not altered the wording of any of the questions on this form.

ICMJE DISCLOSURE FORM

Date: March 4, 2025

Your Name: Temidayo Olutayo Omobowale

Manuscript Title: Apigenin mitigates oxidative stress, neuroinflammation, and cognitive impairment but enhances learning and memory in aluminum chloride-induced neurotoxicity in rats

Manuscript Number (if known): ADJ-D-24-02699R1

In the interest of transparency, we ask you to disclose all relationships/activities/interests listed below that are related to the content of your manuscript. "Related" means any relation with for-profit or not-for-profit third parties whose interests may be affected by the content of the manuscript. Disclosure represents a commitment to transparency and does not necessarily indicate a bias. If you are in doubt about whether to list a relationship/activity/interest, it is preferable that you do so.

The author's relationships/activities/interests should be defined broadly. For example, if your manuscript pertains to the epidemiology of hypertension, you should declare all relationships with manufacturers of antihypertensive medication, even if that medication is not mentioned in the manuscript.

In item #1 below, report all support for the work reported in this manuscript without time limit. For all other items, the time frame for disclosure is the past 36 months.

		Name all entities with whom you have this relationship or indicate none (add rows as needed)	Specifications/Comments (e.g., if payments were made to you or to your institution)
**Time frame: Since the initial planning of the work**			
1	All support for the present manuscript (e.g., funding, provision of study materials, medical writing, article processing charges, etc.) **No time limit for this item.**	☐ None<br>None	None<br>Click the tab key to add additional rows.
**Time frame: past 36 months**			
**2**	Grants or contracts from any entity (if not indicated in item #1 above).	☐ **None**<br>None	None
**3**	Royalties or licenses	☐ **None**<br>None	None

		Name all entities with whom you have this relationship or indicate none (add rows as needed)	Specifications/Comments (e.g., if payments were made to you or to your institution)
**4**	Consulting fees	☐ **None**	
		None	None
**5**	Payment or honoraria for lectures, presentations, speakers bureaus, manuscript writing or educational events	☐ **None**	
		None	None
**6**	Payment for expert testimony	☐ **None**	
		None	None
**7**	Support for attending meetings and/or travel	☐ **None**	
		None	None
**8**	Patents planned, issued or pending	☐ **None**	
		None	None
**9**	Participation on a Data Safety Monitoring Board or Advisory Board	☐ **None**	
		None	None
**10**	Leadership or fiduciary role in other board, society, committee or advocacy group, paid or unpaid	☐ **None**	
		None	None

		Name all entities with whom you have this relationship or indicate none (add rows as needed)	Specifications/Comments (e.g., if payments were made to you or to your institution)
**11**	Stock or stock options	☐ **None**	
		None	None
**12**	Receipt of equipment, materials, drugs, medical writing, gifts or other services	☐ **None**	
		None	None
**13**	Other financial or non-financial interests	☐ **None**	
		None	None

Please place an "X" next to the following statement to indicate your agreement:

X I certify that I have answered every question and have not altered the wording of any of the questions on this form.

ICMJE DISCLOSURE FORM

**Date:**	March 4, 2025
**Your Name:**	Charles Etang Onukak
**Manuscript Title:**	Apigenin mitigates oxidative stress, neuroinflammation, and cognitive impairment but enhances learning and memory in aluminum chloride-induced neurotoxicity in rats
**Manuscript Number (if known):**	ADJ-D-24-02699R1

In the interest of transparency, we ask you to disclose all relationships/activities/interests listed below that are related to the content of your manuscript. "Related" means any relation with for-profit or not-for-profit third parties whose interests may be affected by the content of the manuscript. Disclosure represents a commitment to transparency and does not necessarily indicate a bias. If you are in doubt about whether to list a relationship/activity/interest, it is preferable that you do so.

The author's relationships/activities/interests should be defined broadly. For example, if your manuscript pertains to the epidemiology of hypertension, you should declare all relationships with manufacturers of antihypertensive medication, even if that medication is not mentioned in the manuscript.

In item #1 below, report all support for the work reported in this manuscript without time limit. For all other items, the time frame for disclosure is the past 36 months.

		**Name all entities with whom you have this relationship or indicate none (add rows as needed)**	**Specifications/Comments (e.g., if payments were made to you or to your institution)**
**Time frame: Since the initial planning of the work**			
1	All support for the present manuscript (e.g., funding, provision of study materials, medical writing, article processing charges, etc.) **No time limit for this item.**	☐ None	
		None	None
			Click the tab key to add additional rows.
**Time frame: past 36 months**			
**2**	Grants or contracts from any entity (if not indicated in item #1 above).	☐ **None**	
		None	None
**3**	Royalties or licenses	☐ **None**	
		None	None

		Name all entities with whom you have this relationship or indicate none (add rows as needed)	Specifications/Comments (e.g., if payments were made to you or to your institution)
**4**	Consulting fees	☐ **None**	
		None	None
**5**	Payment or honoraria for lectures, presentations, speakers bureaus, manuscript writing or educational events	☐ **None**	
		None	None
**6**	Payment for expert testimony	☐ **None**	
		None	None
**7**	Support for attending meetings and/or travel	☐ **None**	
		None	None
**8**	Patents planned, issued or pending	☐ **None**	
		None	None
**9**	Participation on a Data Safety Monitoring Board or Advisory Board	☐ **None**	
		None	None
**10**	Leadership or fiduciary role in other board, society, committee or advocacy group, paid or unpaid	☐ **None**	
		None	None

		Name all entities with whom you have this relationship or indicate none (add rows as needed)	Specifications/Comments (e.g., if payments were made to you or to your institution)
**11**	Stock or stock options	☐ **None**	
		None	None
**12**	Receipt of equipment, materials, drugs, medical writing, gifts or other services	☐ **None**	
		None	None
**13**	Other financial or non-financial interests	☐ **None**	
		None	None

Please place an "X" next to the following statement to indicate your agreement:

X I certify that I have answered every question and have not altered the wording of any of the questions on this form.

ICMJE DISCLOSURE FORM

Date: March 4, 2025

Your Name: Ademola Adetokunbo Oyagbemi

Manuscript Title: Apigenin mitigates oxidative stress, neuroinflammation, and cognitive impairment but enhances learning and memory in aluminum chloride-induced neurotoxicity in rats

Manuscript Number (if known): ADJ-D-24-02699R1

In the interest of transparency, we ask you to disclose all relationships/activities/interests listed below that are related to the content of your manuscript. "Related" means any relation with for-profit or not-for-profit third parties whose interests may be affected by the content of the manuscript. Disclosure represents a commitment to transparency and does not necessarily indicate a bias. If you are in doubt about whether to list a relationship/activity/interest, it is preferable that you do so.

The author's relationships/activities/interests should be defined broadly. For example, if your manuscript pertains to the epidemiology of hypertension, you should declare all relationships with manufacturers of antihypertensive medication, even if that medication is not mentioned in the manuscript.

In item #1 below, report all support for the work reported in this manuscript without time limit. For all other items, the time frame for disclosure is the past 36 months.

		**Name all entities with whom you have this relationship or indicate none (add rows as needed)**	**Specifications/Comments (e.g., if payments were made to you or to your institution)**
		**Time frame: Since the initial planning of the work**	
1	All support for the present manuscript (e.g., funding, provision of study materials, medical writing, article processing charges, etc.) **No time limit for this item.**	☐ None	
		None	None
			Click the tab key to add additional rows.
		**Time frame: past 36 months**	
**2**	Grants or contracts from any entity (if not indicated in item #1 above).	☐ **None**	
		None	None
**3**	Royalties or licenses	☐ **None**	
		None	None

<table>
<tr><th></th><th></th><th>Name all entities with whom you have this relationship or indicate none (add rows as needed)</th><th>Specifications/Comments (e.g., if payments were made to you or to your institution)</th></tr>
<tr><td>4</td><td>Consulting fees</td><td>☐ None
None</td><td>None</td></tr>
<tr><td>5</td><td>Payment or honoraria for lectures, presentations, speakers bureaus, manuscript writing or educational events</td><td>☐ None
None</td><td>None</td></tr>
<tr><td>6</td><td>Payment for expert testimony</td><td>☐ None
None</td><td>None</td></tr>
<tr><td>7</td><td>Support for attending meetings and/or travel</td><td>☐ None
None</td><td>None</td></tr>
<tr><td>8</td><td>Patents planned, issued or pending</td><td>☐ None
None</td><td>None</td></tr>
<tr><td>9</td><td>Participation on a Data Safety Monitoring Board or Advisory Board</td><td>☐ None
None</td><td>None</td></tr>
<tr><td>10</td><td>Leadership or fiduciary role in other board, society, committee or advocacy group, paid or unpaid</td><td>☐ None
None</td><td>None</td></tr>
</table>

		Name all entities with whom you have this relationship or indicate none (add rows as needed)	Specifications/Comments (e.g., if payments were made to you or to your institution)
**11**	Stock or stock options	☐ **None**	
		None	None
**12**	Receipt of equipment, materials, drugs, medical writing, gifts or other services	☐ **None**	
		None	None
**13**	Other financial or non-financial interests	☐ **None**	
		None	None

Please place an "X" next to the following statement to indicate your agreement:

X I certify that I have answered every question and have not altered the wording of any of the questions on this form.

ICMJE DISCLOSURE FORM

Date: March 4, 2025

Your Name: Taiwo Olaide Oyagbemi

Manuscript Title: Apigenin mitigates oxidative stress, neuroinflammation, and cognitive impairment but enhances learning and memory in aluminum chloride-induced neurotoxicity in rats

Manuscript Number (if known): ADJ-D-24-02699R1

In the interest of transparency, we ask you to disclose all relationships/activities/interests listed below that are related to the content of your manuscript. "Related" means any relation with for-profit or not-for-profit third parties whose interests may be affected by the content of the manuscript. Disclosure represents a commitment to transparency and does not necessarily indicate a bias. If you are in doubt about whether to list a relationship/activity/interest, it is preferable that you do so.

The author's relationships/activities/interests should be defined broadly. For example, if your manuscript pertains to the epidemiology of hypertension, you should declare all relationships with manufacturers of antihypertensive medication, even if that medication is not mentioned in the manuscript.

In item #1 below, report all support for the work reported in this manuscript without time limit. For all other items, the time frame for disclosure is the past 36 months.

		**Name all entities with whom you have this relationship or indicate none (add rows as needed)**	**Specifications/Comments (e.g., if payments were made to you or to your institution)**
**Time frame: Since the initial planning of the work**			
1	All support for the present manuscript (e.g., funding, provision of study materials, medical writing, article processing charges, etc.) **No time limit for this item.**	☐ None<br>None	None<br><br>Click the tab key to add additional rows.
**Time frame: past 36 months**			
**2**	Grants or contracts from any entity (if not indicated in item #1 above).	☐ **None**<br>None	None
**3**	Royalties or licenses	☐ **None**<br>None	None

		Name all entities with whom you have this relationship or indicate none (add rows as needed)	Specifications/Comments (e.g., if payments were made to you or to your institution)
**4**	Consulting fees	☐ **None**	
		None	None
**5**	Payment or honoraria for lectures, presentations, speakers bureaus, manuscript writing or educational events	☐ **None**	
		None	None
**6**	Payment for expert testimony	☐ **None**	
		None	None
**7**	Support for attending meetings and/or travel	☐ **None**	
		None	None
**8**	Patents planned, issued or pending	☐ **None**	
		None	None
**9**	Participation on a Data Safety Monitoring Board or Advisory Board	☐ **None**	
		None	None
**10**	Leadership or fiduciary role in other board, society, committee or advocacy group, paid or unpaid	☐ **None**	
		None	None

		Name all entities with whom you have this relationship or indicate none (add rows as needed)	Specifications/Comments (e.g., if payments were made to you or to your institution)
**11**	Stock or stock options	☐ **None**	
		None	None
**12**	Receipt of equipment, materials, drugs, medical writing, gifts or other services	☐ **None**	
		None	None
**13**	Other financial or non-financial interests	☐ **None**	
		None	None

Please place an "X" next to the following statement to indicate your agreement:

X I certify that I have answered every question and have not altered the wording of any of the questions on this form.

ICMJE DISCLOSURE FORM

**Date:**	March 4, 2025
**Your Name:**	Adebowale Bernard Saba
**Manuscript Title:**	Apigenin mitigates oxidative stress, neuroinflammation, and cognitive impairment but enhances learning and memory in aluminum chloride-induced neurotoxicity in rats
**Manuscript Number (if known):**	ADJ-D-24-02699R1

In the interest of transparency, we ask you to disclose all relationships/activities/interests listed below that are related to the content of your manuscript. "Related" means any relation with for-profit or not-for-profit third parties whose interests may be affected by the content of the manuscript. Disclosure represents a commitment to transparency and does not necessarily indicate a bias. If you are in doubt about whether to list a relationship/activity/interest, it is preferable that you do so.

The author's relationships/activities/interests should be defined broadly. For example, if your manuscript pertains to the epidemiology of hypertension, you should declare all relationships with manufacturers of antihypertensive medication, even if that medication is not mentioned in the manuscript.

In item #1 below, report all support for the work reported in this manuscript without time limit. For all other items, the time frame for disclosure is the past 36 months.

		**Name all entities with whom you have this relationship or indicate none (add rows as needed)**	**Specifications/Comments (e.g., if payments were made to you or to your institution)**
**Time frame: Since the initial planning of the work**			
1	All support for the present manuscript (e.g., funding, provision of study materials, medical writing, article processing charges, etc.) **No time limit for this item.**	☐ None	
		None	None
			Click the tab key to add additional rows.
**Time frame: past 36 months**			
**2**	Grants or contracts from any entity (if not indicated in item #1 above).	☐ **None**	
		None	None
**3**	Royalties or licenses	☐ **None**	
		None	None

		Name all entities with whom you have this relationship or indicate none (add rows as needed)	Specifications/Comments (e.g., if payments were made to you or to your institution)
**4**	Consulting fees	☐ **None**	
		None	None
**5**	Payment or honoraria for lectures, presentations, speakers bureaus, manuscript writing or educational events	☐ **None**	
		None	None
**6**	Payment for expert testimony	☐ **None**	
		None	None
**7**	Support for attending meetings and/or travel	☐ **None**	
		None	None
**8**	Patents planned, issued or pending	☐ **None**	
		None	None
**9**	Participation on a Data Safety Monitoring Board or Advisory Board	☐ **None**	
		None	None
**10**	Leadership or fiduciary role in other board, society, committee or advocacy group, paid or unpaid	☐ **None**	
		None	None

		Name all entities with whom you have this relationship or indicate none (add rows as needed)	Specifications/Comments (e.g., if payments were made to you or to your institution)
**11**	Stock or stock options	☐ **None**	
		None	None
**12**	Receipt of equipment, materials, drugs, medical writing, gifts or other services	☐ **None**	
		None	None
**13**	Other financial or non-financial interests	☐ **None**	
		None	None

Please place an "X" next to the following statement to indicate your agreement:

X I certify that I have answered every question and have not altered the wording of any of the questions on this form.

ICMJE DISCLOSURE FORM

Date: March 4, 2025

Your Name: Adeola Temitope Salami

Manuscript Title: Apigenin mitigates oxidative stress, neuroinflammation, and cognitive impairment but enhances learning and memory in aluminum chloride-induced neurotoxicity in rats

Manuscript Number (if known): ADJ-D-24-02699R1

In the interest of transparency, we ask you to disclose all relationships/activities/interests listed below that are related to the content of your manuscript. "Related" means any relation with for-profit or not-for-profit third parties whose interests may be affected by the content of the manuscript. Disclosure represents a commitment to transparency and does not necessarily indicate a bias. If you are in doubt about whether to list a relationship/activity/interest, it is preferable that you do so.

The author's relationships/activities/interests should be defined broadly. For example, if your manuscript pertains to the epidemiology of hypertension, you should declare all relationships with manufacturers of antihypertensive medication, even if that medication is not mentioned in the manuscript.

In item #1 below, report all support for the work reported in this manuscript without time limit. For all other items, the time frame for disclosure is the past 36 months.

		**Name all entities with whom you have this relationship or indicate none (add rows as needed)**	**Specifications/Comments (e.g., if payments were made to you or to your institution)**
**Time frame: Since the initial planning of the work**			
1	All support for the present manuscript (e.g., funding, provision of study materials, medical writing, article processing charges, etc.) **No time limit for this item.**	☐ None<br>None	None
			Click the tab key to add additional rows.
**Time frame: past 36 months**			
**2**	Grants or contracts from any entity (if not indicated in item #1 above).	☐ **None**<br>None	None
**3**	Royalties or licenses	☐ **None**<br>None	None

		Name all entities with whom you have this relationship or indicate none (add rows as needed)	Specifications/Comments (e.g., if payments were made to you or to your institution)
**4**	Consulting fees	☐ **None**	
		None	None
**5**	Payment or honoraria for lectures, presentations, speakers bureaus, manuscript writing or educational events	☐ **None**	
		None	None
**6**	Payment for expert testimony	☐ **None**	
		None	None
**7**	Support for attending meetings and/or travel	☐ **None**	
		None	None
**8**	Patents planned, issued or pending	☐ **None**	
		None	None
**9**	Participation on a Data Safety Monitoring Board or Advisory Board	☐ **None**	
		None	None
**10**	Leadership or fiduciary role in other board, society, committee or advocacy group, paid or unpaid	☐ **None**	
		None	None

		Name all entities with whom you have this relationship or indicate none (add rows as needed)	Specifications/Comments (e.g., if payments were made to you or to your institution)
**11**	Stock or stock options	☐ **None**	
		None	None
**12**	Receipt of equipment, materials, drugs, medical writing, gifts or other services	☐ **None**	
		None	None
**13**	Other financial or non-financial interests	☐ **None**	
		None	None

Please place an "X" next to the following statement to indicate your agreement:

X I certify that I have answered every question and have not altered the wording of any of the questions on this form.

ICMJE DISCLOSURE FORM

Date: March 4, 2025

Your Name: Momoh Audu Yakubu

Manuscript Title: Apigenin mitigates oxidative stress, neuroinflammation, and cognitive impairment but enhances learning and memory in aluminum chloride-induced neurotoxicity in rats

Manuscript Number (if known): ADJ-D-24-02699R1

In the interest of transparency, we ask you to disclose all relationships/activities/interests listed below that are related to the content of your manuscript. "Related" means any relation with for-profit or not-for-profit third parties whose interests may be affected by the content of the manuscript. Disclosure represents a commitment to transparency and does not necessarily indicate a bias. If you are in doubt about whether to list a relationship/activity/interest, it is preferable that you do so.

The author's relationships/activities/interests should be defined broadly. For example, if your manuscript pertains to the epidemiology of hypertension, you should declare all relationships with manufacturers of antihypertensive medication, even if that medication is not mentioned in the manuscript.

In item #1 below, report all support for the work reported in this manuscript without time limit. For all other items, the time frame for disclosure is the past 36 months.

		**Name all entities with whom you have this relationship or indicate none (add rows as needed)**	**Specifications/Comments (e.g., if payments were made to you or to your institution)**
**Time frame: Since the initial planning of the work**			
1	All support for the present manuscript (e.g., funding, provision of study materials, medical writing, article processing charges, etc.) **No time limit for this item.**	☐ None<br>None	None<br>Click the tab key to add additional rows.
**Time frame: past 36 months**			
**2**	Grants or contracts from any entity (if not indicated in item #1 above).	☐ **None**<br>None	None
**3**	Royalties or licenses	☐ **None**<br>None	None

		Name all entities with whom you have this relationship or indicate none (add rows as needed)	Specifications/Comments (e.g., if payments were made to you or to your institution)
**4**	Consulting fees	☐ **None**	
		None	None
**5**	Payment or honoraria for lectures, presentations, speakers bureaus, manuscript writing or educational events	☐ **None**	
		None	None
**6**	Payment for expert testimony	☐ **None**	
		None	None
**7**	Support for attending meetings and/or travel	☐ **None**	
		None	None
**8**	Patents planned, issued or pending	☐ **None**	
		None	None
**9**	Participation on a Data Safety Monitoring Board or Advisory Board	☐ **None**	
		None	None
**10**	Leadership or fiduciary role in other board, society, committee or advocacy group, paid or unpaid	☐ **None**	
		None	None

		Name all entities with whom you have this relationship or indicate none (add rows as needed)	Specifications/Comments (e.g., if payments were made to you or to your institution)
**11**	Stock or stock options	☐ **None**	
		None	None
**12**	Receipt of equipment, materials, drugs, medical writing, gifts or other services	☐ **None**	
		None	None
**13**	Other financial or non-financial interests	☐ **None**	
		None	None

Please place an "X" next to the following statement to indicate your agreement:

X I certify that I have answered every question and have not altered the wording of any of the questions on this form.
